# Swartkrans *Paranthropus* and Sterkfontein *Australopithecus* from southern Africa had different locomotor repertoires

**DOI:** 10.1073/pnas.2532193123

**Published:** 2026-05-11

**Authors:** Marine Cazenave, Annalisa Pietrobelli, Andrea Luková, Sebastian Bachmann, Matthew V. Caruana, Ronald J. Clarke, Christopher J. Dunmore, Ashley S. Hammond, Jason L. Heaton, A. J. Heile, Jakobus Hoffman, Kathleen Kuman, Dieter H. Pahr, Christopher M. Smith, Dominic Stratford, Alexander Synek, Zewdi J. Tsegai, Tracy L. Kivell, Travis Rayne Pickering, Matthew M. Skinner

**Affiliations:** ^a^ Department of Human Origins, Max Planck Institute for Evolutionary Anthropology, Leipzig 04103, Germany; ^b^ Division of Anthropology, American Museum of Natural History, New York, NY 10024-5102; ^c^ Department of Anatomy, Faculty of Health Sciences, University of Pretoria, Pretoria 0084, South Africa; ^d^ Department of Anthropology, University of West Bohemia in Pilsen, Pilsen 301 00, Czech Republic; ^e^ Institute of Lightweight Design and Structural Biomechanics, Technischen Universität Wien, Vienna 1060, Austria; ^f^ Evolutionary Studies Institute, University of the Witwatersrand, Johannesburg 2017, South Africa; ^g^ Palaeo-Research Institute, University of Johannesburg, Johannesburg 2006, South Africa; ^h^ School of Biosciences, University of Kent, Canterbury CT2 7NZ, United Kingdom; ^i^Institut Català de Paleontologia Miquel Crusafont, Universitat Autònoma de Barcelona, Barcelona 08193, Spain; ^j^ Institució Catalana de Recerca i Estudis Avançats, Barcelona 08010, Spain; ^k^ Department of Biology, University of Alabama at Birmingham, Birmingham, AL 35205; ^l^ Department of Anthropology, University of Wisconsin, Madison, WI 53706; ^m^ South African Nuclear Energy Corporation, Pelindaba 0240, South Africa; ^n^ School of Geography, Archaeology and Environmental Studies, University of the Witwatersrand, Johannesburg 2000, South Africa; ^o^ Department of Biology, Fairfield University, Fairfield, CT 06824; ^p^ New York Consortium in Evolutionary Primatology, New York, NY 10024; ^q^ Department of Anthropology, Stony Brook University, Stony Brook, NY 11794; ^r^ Department of Organismal Biology and Anatomy, University of Chicago, Chicago, IL 60637; ^s^ Department of Archaeogenetics, Max Planck Institute for Evolutionary Anthropology, Leipzig 04103, Germany

## Abstract

Southern African hominin fossils traditionally attributed to *Paranthropus robustus* and *Australopithecus africanus* are differentiated from each other by their dentition and cranial architecture, but their postcranial anatomy has typically been regarded functionally as broadly similar (i.e., terrestrial bipedalism with some degree of arboreal locomotion). Testing the hypothesis of a similar locomotor repertoire between these two taxa has been complicated by a lack of postcranial fossils attributable to *P. robustus*. Here, we detail our comparative examination of the internal bone anatomy of a recently described c. 1.8 Ma *P. robustus* articulating femur and tibia, which suggests distinct patterns of joint loading and locomotor behavior. Our analysis of cortical bone in the hip and trabecular bone in the ankle, knee, and hip joints suggests that *P*. *robustus* habitually adopted a high frequency of ankle, knee, and hip flexion, as required for climbing, while *Australopithecus* from a c. 3.4 Ma level at the site of Sterkfontein displays a more modern human-like structural pattern across the lower limb joints consistent with comparatively more frequent terrestrial bipedalism. These results reveal that geologically younger *P*. *robustus* likely used arboreality more frequently than older Sterkfontein *Australopithecus*. Together with differences in masticatory behavior, this line of evidence indicates that these two hominin taxa occupied distinct ecological niches.

## Significance

Southern African *Paranthropus robustus* and Sterkfontein *Australopithecus* are traditionally distinguished by their dentition and cranial architecture, while postcranial anatomy has often been assumed to be similar, reflecting a combination of terrestrial bipedalism and some arboreal climbing. To test this assumption, we examined internal structure of a recently discovered *P*. *robustus* articulating femur and tibia, focusing on cortical and trabecular bone of the ankle, knee, and hip. We show clear functional differences: *P*. *robustus* exhibits features indicative of joint postures likely associated with frequent climbing, whereas Sterkfontein *Australopithecus* shows a pattern consistent with comparatively more frequent terrestrial bipedalism. Thus, the younger *P*. *robustus* likely retained more arboreal behaviors than the older Sterkfontein *Australopithecus*, consistent with distinct locomotor repertoires and ecological niches.

In 1938, the first *Paranthropus robustus* was discovered at Kromdraai within the Cradle of Humankind, South Africa (1). *P. robustus* has been differentiated from fossils traditionally attributed to *Australopithecus africanus*, also found in the same region, by its specialized dentition with enlarged premolars and molars relative to smaller canines and incisors, and the associated cranial morphology consistent with well-developed chewing musculature (2). Additional discoveries and analyses have confirmed that the geologically younger *P*. *robustus* [2.22 ± 0.09 to 0.96 + 0.09 million years (Ma)] (3) and the older *A. africanus* [c. 3.4 to 3.5 Ma (4) (contra (5) with dating of c. 2.61 to 2.07 Ma)] also differed in dental microwear and enamel thickness (reviewed in ref. 6). It has been suggested that *P*. *robustus* consumed hard and brittle food items while *A*. *africanus* preferred to eat more fibrous foods, but that both taxa likely had, at least during some periods, variable and overlapping diets (7) (but see refs. 8 and 9). This hypothesis of differing primary diets is also supported by isotopic analyses (10–13).

In contrast to the considerable body of research on the functional and taxonomic significance of the southern African *Paranthropus* and Sterkfontein M4 *Australopithecus* craniodental morphology (14), quantitative comparative studies of their postcranial fossils are limited. In the early 1970s, Robinson (15), noted that *P. robustus* had a long ischium and features of its foot morphology suggesting that it spent some time in the trees and was not so bipedally efficient on the ground as was *Homo africanus* (i.e., *A*. *africanus*). Subsequently, a few studies, notably on lower limb fossils from Sterkfontein and Swartkrans, discuss some degree of morphological and, thus locomotor diversity among early hominins and between Sterkfontein *Australopithecus* and the Swartkrans *Paranthropus* (*SI Appendix*, Fig. S1) but it has been largely assumed that both taxa were similar postcranially [(16, 17); see references in ref. 18]. The preserved lower limb morphologies of these taxa indicate bipedalism as the terrestrial locomotion mode (19, 20), while aspects of their upper limb morphologies indicate some degree of arboreality (21, 22). However, the rare association in the southern African fossil record of postcranial elements with taxonomically diagnostic craniodental materials has complicated interpretations of the nature, significance, and implications of any morphological differences (23, 24).

The internal bone structure of hominin lower limb bones offers insights on locomotion because cortical and trabecular bone model and remodel in response to loads incurred during life (reviewed in ref. 25, and *SI Appendix*, Text S1 and *SI Appendix*, II). Experimental studies of animal lower limb internal bone structure have shown that, within a given joint, regions of high trabecular density correspond to areas of relative high loading conditions (i.e., magnitude and frequency of stress), while strut orientation reflects the direction of loading during locomotion (26, 27 and *SI Appendix*, II). Applying these experimental results to hominin fossils allows inference of joint loading and, in turn, locomotor behavior during life. For example, analysis of the trabecular arrangement in two femoral heads from Sterkfontein—StW 522 [Member 4 (M4); ca 3.4-3.5 Ma (4)] and StW 311 [Member 5 (M5); in undated deposits with some solution pockets (4)]—revealed different patterns at the hip joint despite their similar external shape (28). However, while the fossil from Member 4 is traditionally attributed to *A. africanus*, it is unclear whether the specimen from Member 5 represents *P*. *robustus* or early *Homo*. Although there are some aspects of the *P*. *robustus* proximal femur that distinguish it from other taxa (*SI Appendix*, Fig. S1), taxonomically diagnostic craniodental remains of *P*. *robustus* almost invariably co-occur with early *Homo* fossils, making it difficult to attribute isolated postcranial fossils to either taxon (29, 30).

A recently described articulating tibia (SWT1/HR-2c) and femur (SWT1/HR-2b) from Swartkrans Member 1 (M1) can be attributed to *P*. *robustus* based on the external morphology of the proximal femur (31). Thus, these specimens allow investigation of the internal bone structure of the lower limb of *P*. *robustus*. To test the hypothesis that *P*. *robustus* and Sterkfontein M4 *Australopithecus* engaged in similar locomotor behaviors, we compared the internal bone structure of the distal tibia of SWT1/HR-2c and the distal femur of SWT1/HR-2b, along with additional fossil proximal femora from Swartkrans M1 (n = 3), to several *Australopithecus* tibiae and femora from Sterkfontein M4 (n = 5). Fossils found at Sterkfontein M4 have typically been assigned to *A*. *africanus*, but following the proposal by Clarke (32) that a second *Australopithecus* species is represented in that geological unit, we simply use the term ‘Sterkfontein M4 *Australopithecus*’ from hereon to refer all Sterkfontein M4 remains (*SI Appendix*, Fig. S1). We performed 3D quantitative analyses of the trabecular structure of the distal tibia (ankle) and femur (knee) and the femoral head (hip), as well as quantifying cortical bone thickness in the femoral neck, in 10 fossil hominin specimens within the broader comparative context of modern humans (*Homo sapiens*) and extant African apes (*Pan troglodytes* and *Gorilla gorilla*) (*SI* *Appendix*, Tables S1 and S2).

## Results

### Trabecular Bone Patterning of the Ankle Joint.

Following automatic segmentation of bone tissue (*Materials and Methods*), we quantified and statistically compared trabecular bone volume fraction (i.e., the ratio of bone volume to total volume [BV/TV]) across the entire epiphysis using canonical holistic morphometric analysis (cHMA) (33). We standardized all raw BV/TV values (*Materials and Methods*) to account for taxon-specific differences (e.g., systemic differences in overall trabecular bone density) and thus compare relative BV/TV (rBV/TV) across our extant and fossil sample, thus facilitating examination of between-species differences in trabecular bone density (*SI Appendix*, II). This innovative approach allows for holistic and homologous statistical comparisons [here, principal component analyses (PCA), canonical variate analyses (CVA), typicality probabilities, and multivariate ANOVA (MANOVA)] of trabecular bone patterning throughout each epiphysis, while accounting for variation in bone shape. Differences in rBV/TV distribution are expected to reflect differences in joint loading during habitual behaviors across taxa. This approach has previously revealed taxon-specific patterns of trabecular structure that indicate differences in joint loading consistent with habitual behaviors in extant taxa, which are not captured by single volume-of-interest analyses (25, 34, 35), thus providing more robust inferences of hominin behaviors in the past. A major advantage of the cHMA approach is that for incompletely preserved fossil specimens (e.g., the distal tibiae in our sample) we can still register them to the canonical model allowing selective analysis of specific cells based only on those regions in which trabecular bone is sufficiently preserved.

**Fig. 1.**
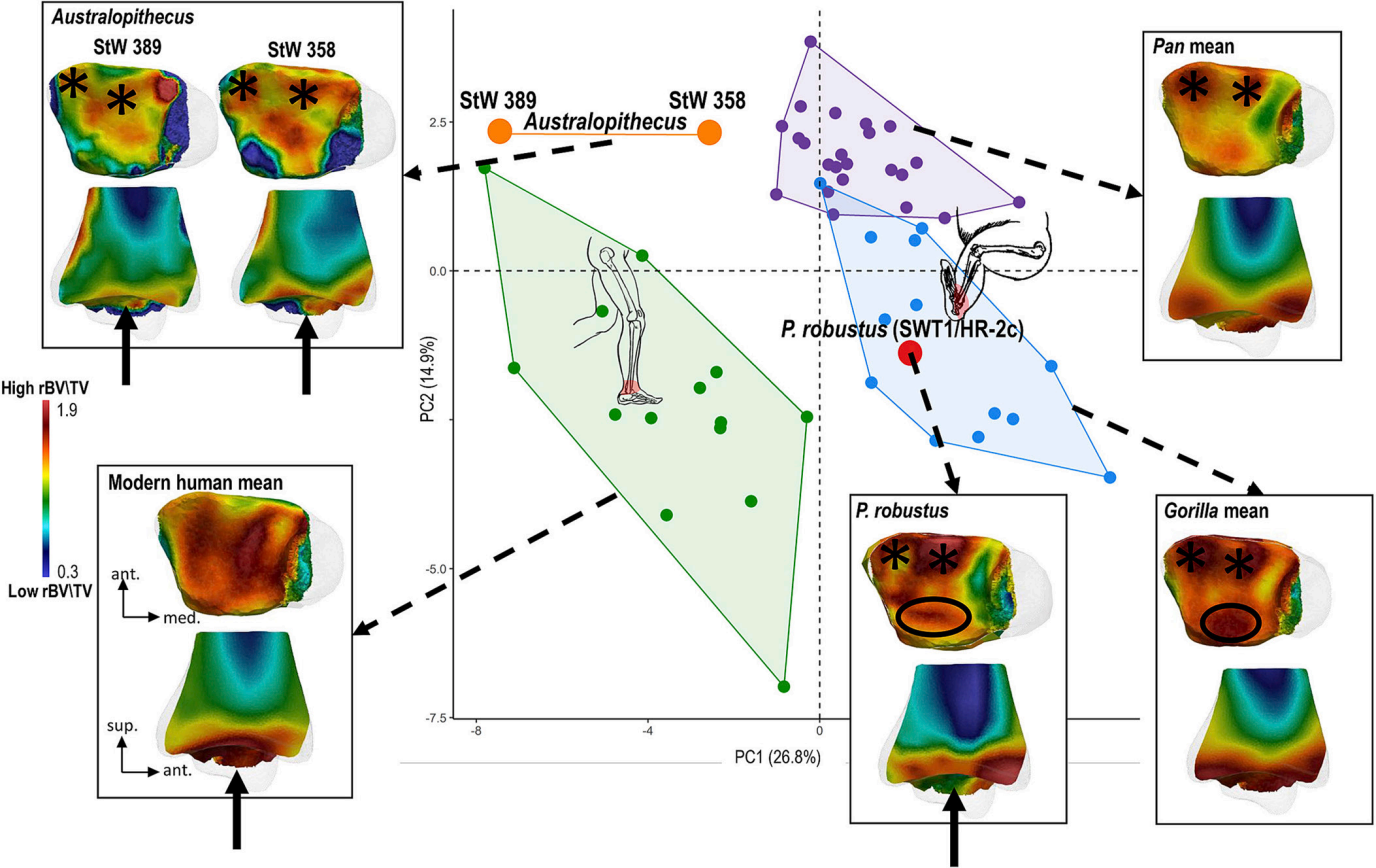
PCA of the trabecular relative bone density (rBV/TV) of the distal tibia of the southern African *P. robustus*, Sterkfontein M4 *Australopithecus,* and the extant comparative sample of modern humans, *Pan,* and *Gorilla* individuals. The first two principal components (PCs) discriminate between modern humans and each African ape. The *P. robustus* specimen from Swartkrans Member 1 (SWT1/HR-2c) falls in the *Gorilla* convex hull and the *Australopithecus* specimens from Sterkfontein Member 4 (StW 358, StW 389) fall in their own morphospace between the modern human and African ape distributions. Trabecular rBV/TV distribution in inferior view (above) and a sagittal midline cross-section (below) is shown, onto the unique canonical mesh (in transparent), for each fossil and as a mean model of all specimens for each extant taxon. All color maps are shown to the same rBV/TV scale, average values are the same across all specimens. Black stars highlight the regions of high rBV/TV concentration along the anterior and anterolateral margins shared by the fossils and African apes. The black circle indicates the posterior rBV/TV reinforcement shared between *Paranthropus* and African apes. The black arrow marks the central bone reinforcement shared by the fossils and modern humans.

The ankle (tibiotalar joint) is the lower limb joint nearest to the substrate and the primary point of force transfer and stabilization during bipedal locomotion. Both Sterkfontein M4 *Australopithecus* and *P*. *robustus* display a modern human-like shape of the tibial articular surface that provides foot stabilization during bipedal walking as well as reducing the likelihood of joint dislocation (31, 36). The newly discovered tibia SWT1/HR-2c is the first *P*. *robustus* tibia in the fossil record and only the third complete tibia from all of the Plio-Pleistocene sites in southern Africa. In contrast, the Sterkfontein M4 *Australopithecus* distal tibiae StW 358 and StW 389 are less complete, with the latter missing its medial malleolus (*SI Appendix*, Figs. S1 and S2). Thus, the medial malleolar region was excluded from the analysis. Statistical analyses of rBV/TV distribution show that *Australopithecus* distal tibiae align with those of modern humans on PC1 and are closer to *Pan* on PC2 (Fig. 1 and *SI Appendix*, Text S2 and Fig. S3). Both of the Sterkfontein M4 *Australopithecus* specimens reveal a modern human-like central concentration of high rBV/TV on the distal articular surface, consistent with a more stable ankle joint and a more perpendicular load transfer from the talus to the distal tibia (37). Both specimens (particularly StW 358 see *SI Appendix*, Text S3) also show higher rBV/TV at the anterior margin of the distal tibial articulation resembling that of great apes, a pattern likely linked to loading in dorsiflexion (38, 39) (Fig. 1 and *SI Appendix*, Fig. S3 and Table S3). While the *P*. *robustus* specimen shares with the Sterkfontein M4 *Australopithecus* fossils the modern human-like central bone reinforcement and the African ape-like rBV/TV at the anterior margin, in contrast to the Sterkfontein fossils, its distal tibia follows the typical African ape trabecular pattern with also higher rBV/TV on the posterior tibiotalar articular margins as compared with modern humans (Fig. 1 and *SI Appendix*, Text S2, Fig. S3, and Table S3). In African apes, this trabecular distribution is consistent with a more mobile ankle joint loaded with a dorsiflexed, inverted foot (37, 38). Subtle differences are observed between *Pan* and *Gorilla*, where the posterior concentration expands farther toward the subarticular center in *Gorilla* than in *Pan*, likely related, as discussed previously (40), to differences in degree of plantarflexion during vertical climbing. The CVA on the first five PCs confirms these results, with the calculated typicality probability showing a closer affinity of SWT1/HR-2c with the *Gorilla* sample (*P* = 0.62) (*SI Appendix*, Fig. S4).

### Trabecular Bone Patterning of the Knee Joint.

**Fig. 2.**
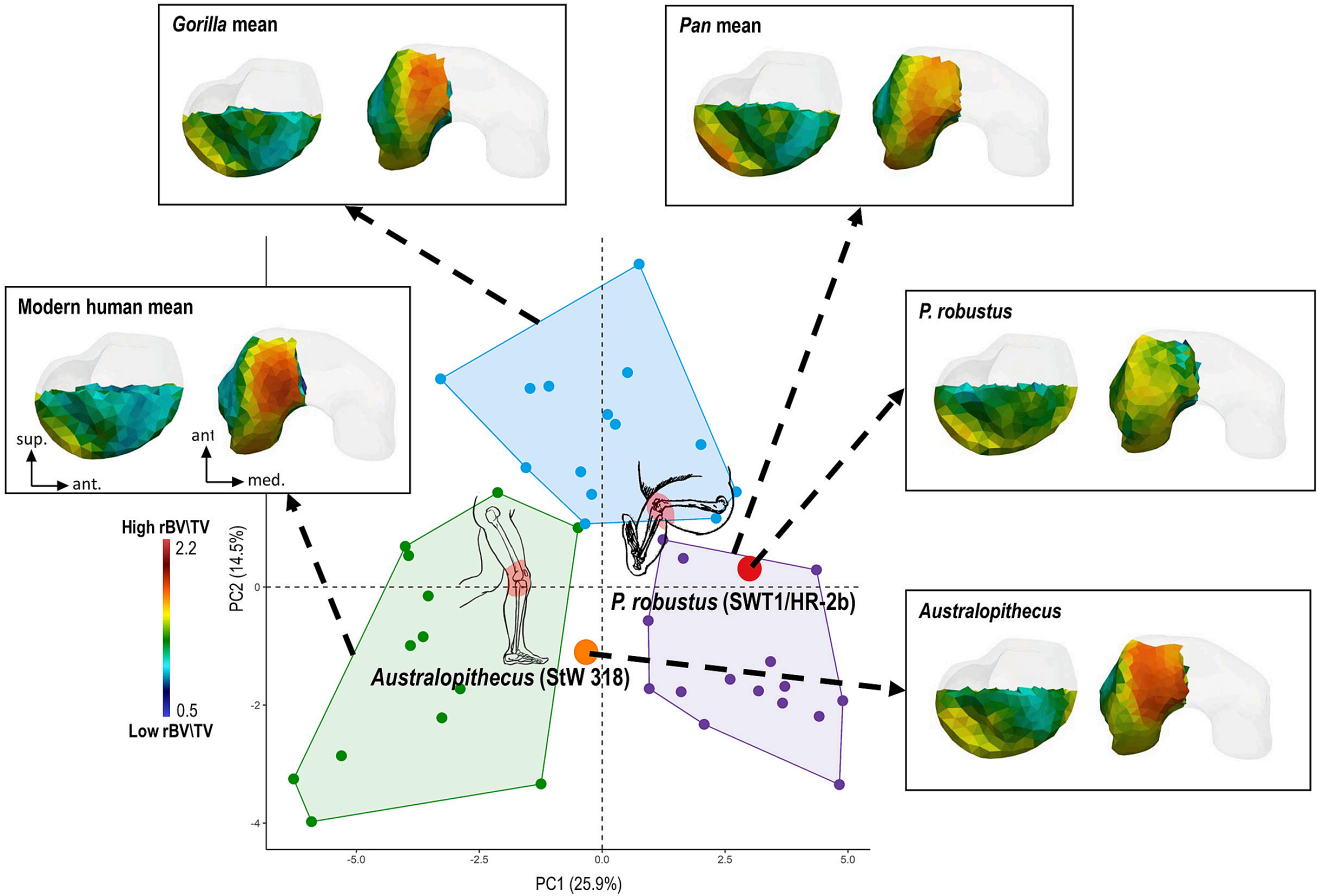
PCA of the trabecular relative bone density (rBV/TV) in the distal femur (lateral condyle) of the southern African *P. robustus*, Sterkfontein M4 *Australopithecus,* and the extant comparative sample of modern humans, *Pan,* and *Gorilla* individuals. The first two PCs discriminate across modern humans, *Pan* and *Gorilla*. *P. robustus* (SWT1/HR-2b) falls in the *Pan* convex hull and the *Australopithecus* specimen (StW 318) falls in its own morphospace between the modern human and *Pan* distributions. Trabecular rBV/TV distribution in sagittal midline cross-section of the lateral condyle (*Left*) and inferior view (*Right*) is shown, onto the unique canonical mesh (in transparent), for each fossil and as a mean model of all specimens for each extant taxon. All color maps are shown to the same rBV/TV scale between 0.5 and 2.2, average values are the same across all specimens.

Like the ankle, the external morphology of the knee of Sterkfontein M4 *Australopithecus* and *P*. *robustus* shows derived modern human-like features (*SI Appendix*, Fig. S1). These characteristics are associated with an extended-knee posture during bipedalism and they have been key evidence for previous arguments that hominins with such derived knee joint morphology no longer retained climbing within their locomotor repertoire (41–43). The newly discovered *P*. *robustus* femur, SWT1/HR-2b, is the first femur of this species to preserve a complete distal epiphysis and the first opportunity to use trabecular bone architecture to infer joint loading in locomotor-related knee biomechanics (31). Although the whole epiphysis is preserved externally, taphonomic modifications mean that we can only examine trabecular bone patterning in the inferior portion of the lateral condyle (*SI Appendix*, Text S4 and Fig. S2). In Sterkfontein M4 *Australopithecus* (StW 318), the highest trabecular bone density is found at the patellar articular surface, a pattern similar to that found in modern humans that reflects compressive loadings during the lateral pull of the patella by the quadriceps tendon during extended-knee bipedalism with a valgus knee angle (Fig. 2 and *SI Appendix*, Fig. S5 and Table S3). The CVA typicality probability is also similar to that of modern humans (*P* = 0.13; *SI Appendix*, Fig. S6). In contrast, the highest bone density in *P*. *robustus* occurs in the most posterior region of the femoral condyle, as in African apes, and in the PCA, *P*. *robustus* falls within the *Pan* distribution (Fig. 2 and *SI Appendix*, Fig. S5 and Table S3), with the CVA calculated typicality probability assigning this specimen to *Pan* (*P* = 0.51; *SI Appendix*, Fig. S5). The high rBV/TV in the posterior region of the condyle likely reflects more frequent loading in knee flexion (34), such as when during climbing the posterior portion of the condyles is in contact with the proximal tibia, generating a high compressive load in this contact zone.

### Trabecular and Cortical Bone Patterning At the Hip Joint.

**Fig. 3.**
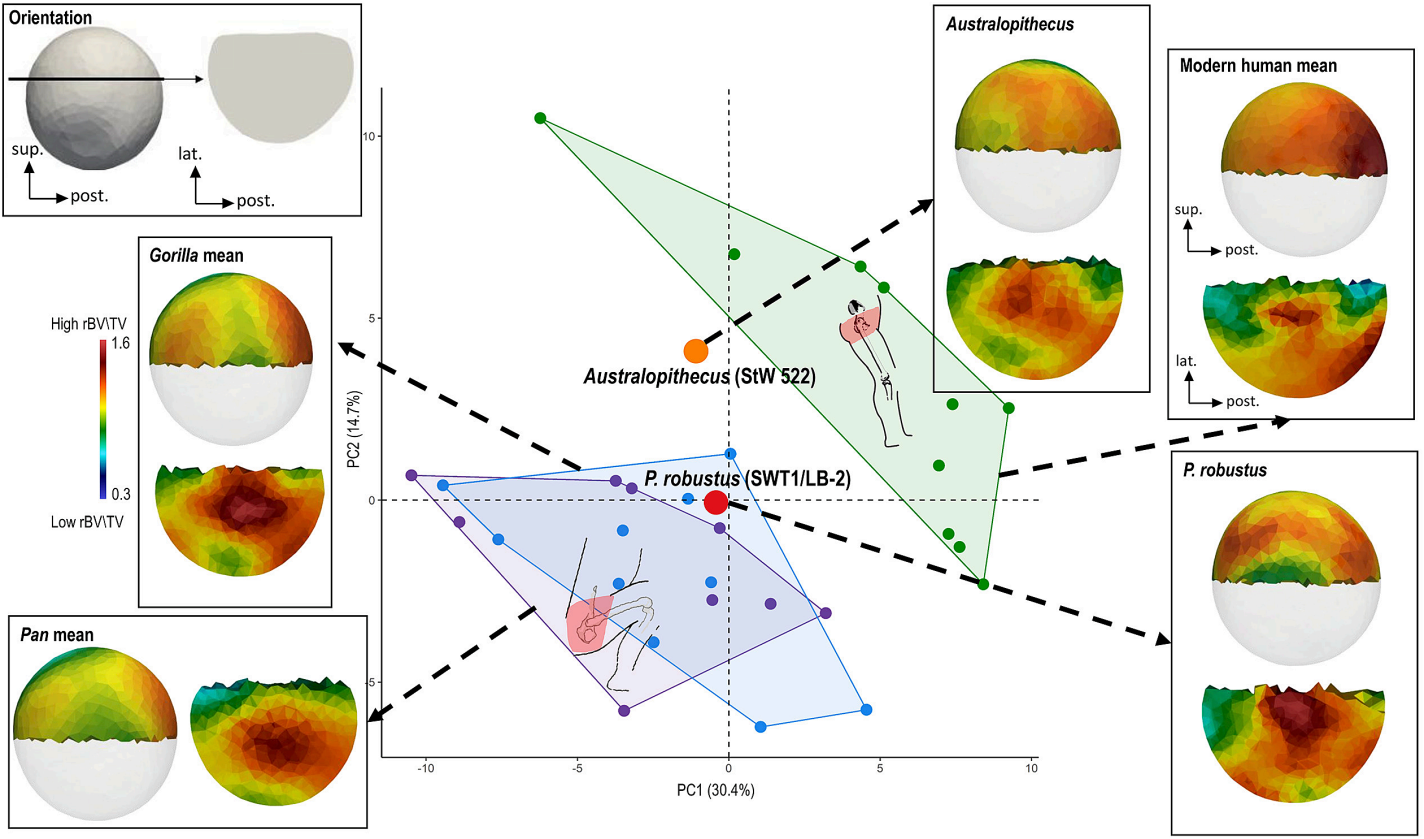
PCA of the trabecular relative bone density (rBV/TV) of the superior portion of the femoral head of the southern African *P. robustus*, Sterkfontein M4 *Australopithecus,* and the extant comparative sample of modern humans, *Pan,* and *Gorilla* individuals. The first two PCs discriminate modern humans from African apes. The *P. robustus* specimen from Swartkrans Member 1 (SWT1/LB-2) falls in the *Gorilla* convex hull and the *Australopithecus* specimen from Sterkfontein Member 4 (StW 522) falls in its own morphospace between the modern human and African ape convex hulls. Trabecular rBV/TV distribution in medial view (*Above*) and a coronal cross-section in superior view (*Below*) is shown onto the unique canonical mesh (in transparent), for each fossil and as a mean model of all specimens for each extant taxon. All color maps are shown to the same rBV/TV scale between 0.3 and 1.6, average values are the same across all specimens.

Trabecular bone distribution in the femoral head and cortical bone thickness distribution around the femoral neck have been extensively studied across primates, including experimental analyses (*SI Appendix*, Text S1). In the *P*. *robustus* proximal femur SWT1/LB-2 from Swartkrans M1 (*SI Appendix*, Text S1), only the superior portion of the head preserves the trabecular structure well enough to be confidently quantified (*SI Appendix*, Fig. S2) and analyzed. In Sterkfontein M4 *Australopithecus* StW 522, rBV/TV in the superior portion of the femoral head shows only a posterior reinforcement as is the case in modern humans where habitual loading occurs in an extended-hip posture. Indeed, the specimen falls within the modern human distribution of the PCA (Fig. 3 and *SI Appendix*, Figs. S7 and S8 and Table S3), and CVA typicality probability assigns it to the modern human sample (*P* = 0.30; *SI Appendix*, Fig. S9). In contrast, the *P*. *robustus* SWT1/LB-2 shows an African ape-like pattern of rBV/TV distribution, with posterior and anterior concentrations of high rBV/TV (*SI Appendix*, Text S5). This specimen falls within the African ape distribution of the PCA (Fig. 3 and *SI Appendix*, Figs. S7 and S8 and Table S3) and the CVA calculated typicality probability is closest to *Gorilla* (*P* = 0.50; *SI Appendix*, Fig. S9). Trabecular reinforcement in the anterior region of the SWT1/LB-2 femoral head results from the contact loadings with the acetabulum during frequent hip flexion, and the posterior reinforcement reflects loading during a more extended hip joint posture (28, 44, 45).

**Fig. 4.**
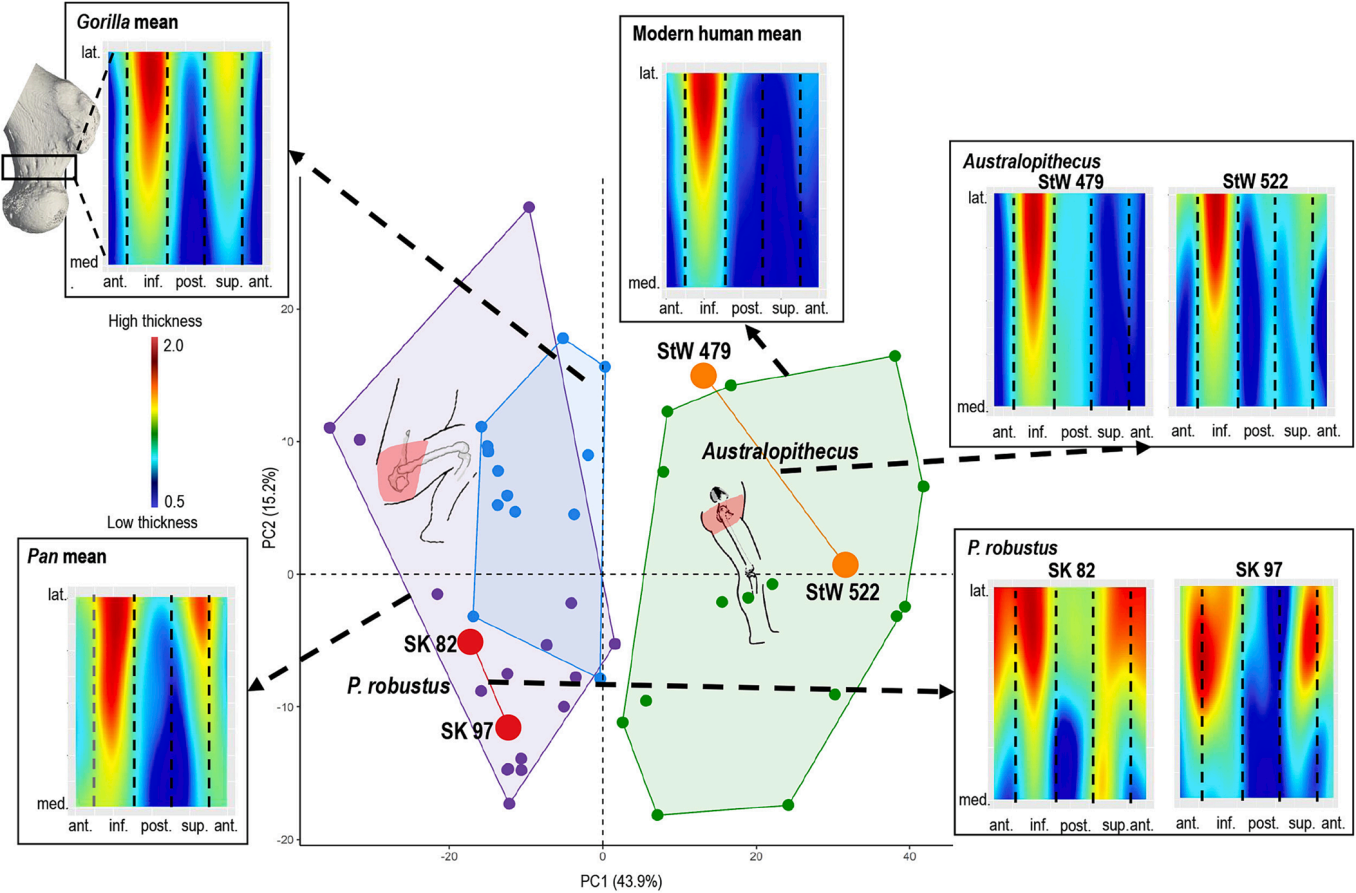
PCA of the relative cortical thickness distribution of the femoral neck of the southern African *P. robustus*, Sterkfontein M4 *Australopithecus,* and the extant comparative sample of modern humans, *Pan,* and *Gorilla* individuals. The first two PCs discriminate modern humans from African apes. The *P. robustus* specimens (SK 82 and SK 97) fall in the *Pan* convex hull and the *Australopithecus* specimens (StW 479 and StW 522) fall in or at the edge of the modern human range of variation. A color map of the “unrolled” relative cortical thickness distribution across the femoral neck is shown for each fossil and as mean map of all specimens for each extant taxon. All cortical color maps are shown to the same scale between 0.5 and 2, average values are the same across all specimens.

The inference that *P*. *robustus* loaded its hip joint in a larger range of postures than do modern humans is also supported when the distribution of standardized cortical thickness in the entire femoral neck is quantified using morphomap (46) (Fig. 4 and *SI Appendix*, Fig. S2). The analysis of the two best-preserved *P*. *robustus* specimens from Swartkrans M1 [SK 82 and SK 97; taphonomic alteration prevents analysis of cortical thickness in SWT1/LB-2 (47)] suggests a different pattern from that of Sterkfontein M4 *Australopithecus* (StW 479 and StW 522) and modern humans (*SI Appendix*, Text S6). The most notable difference is that *P*. *robustus* exhibits thick cortical bone in the superior and inferior portions of the femoral neck. This more homogenous distribution of cortical thickness, which is similar to that of African apes and likely reflects more frequent flexion and abduction at the hip (48) is unlike the modern human-like Sterkfontein M4 *Australopithecus* femora (StW 522 and StW 479) (*SI Appendix*, Figs. S10–S12 and Table S3).

## Discussion

Over the past two decades, new fossil evidence, including two partial skeletons (*Australopithecus* *sediba*) and one almost complete skeleton (StW 573) of *Australopithecus*, in southern Africa (49, 50), the 3.4 Ma old Burtele foot in Ethiopia (51), and the 1.5 Ma footprints in Kenya (52) clearly demonstrate that early hominins did not all move bipedally in the same way. Multiple hominin species with different degrees of arboreal and terrestrial adaptations existed and may have coexisted. Our findings suggest an even more complex picture when considering plastic ecophenotypic aspects of bone structure that reflect how individuals were loading their joints during life. Both *P*. *robustus* from Swartkrans M1 and *Australopithecus* from Sterkfontein M4 share external morphological adaptations to terrestrial bipedalism at the ankle, knee, and hip joints (*SI Appendix*, Fig. S1). However, the cortical and trabecular distributions, at three locations whose structural organization reflects joint locomotor-related loading conditions (*SI Appendix*, Text S1 and *SI Appendix*, II), show distinct patterns between the two taxa across the whole lower limb that, we argue, result from different habitual positional and locomotor behaviors in these hominins (*SI Appendix*, Fig. S13).

The similarities of *P*. *robustus* internal bone structure with that of African apes, particularly in the articulating lower limb bones of the SWT1/HR-2 young adult individual, indicate that *P*. *robustus* routinely flexed and abducted its hip, as well as flexed its knee and ankle joints. This evidence, however, does not imply that *P*. *robustus* was not bipedal. Its external lower limb morphology shows adaptations to bipedalism (*SI Appendix*, Fig. S1) and its internal bone structure also exhibits patterns compatible with extended limb bipedal locomotion as observed in modern humans. For example, trabeculae in the center of distal tibial articular surface are reinforced, reflecting important vertical loading during the stance phase of the bipedal gait. These results are consistent with, and further support, Robinson’s (15) earlier interpretations based on the morphology of the ischium as well as the hand and foot bones, suggesting that although *P*. *robustus* must have had appreciable capability as a biped, it was not as effectively specialized in this direction as was *H. africanus* (sic) (15). Together, these findings reinforce the functional signal identified in more recent morphological analyses documenting differences between *A*. *africanus* and *P*. *robustus* (*SI Appendix*, Fig. S1).

The interpretation that this trabecular pattern reflects dorsi- and plantarflexed ankle joint postures, combined with flexed hip, is grounded in previous analyses of trabecular bone in extant primates that explicitly link internal bone structure to documented joint kinematics in African apes. At the distal tibia (40), the tibiotalar articulation shifts according to ankle position: In dorsiflexion, the talus articulates more anteriorly against the tibial plafond, whereas in plantarflexion, it articulates more posteriorly, concentrating compressive loads along the respective margins (53). A posterior reinforcement of trabecular bone therefore indicates repeated loading in plantarflexed positions (54). Such pronounced plantarflexion occurs during climbing in African apes (peak plantarflexion: −12 ± 10° from a horizontal or neutral ankle position) and is not characteristic of terrestrial locomotion (54, 55). At the femoral head (56), the anterior region of the head comes into contact with the lunate surface of the acetabulum during phases of full hip flexion, the hip can be flexed to a maximum of 25° to 55° in *Pan* and less than 30° in *Gorilla* during vertical climbing [angle between the trunk and the femur; (45, 57)], generating localized compressive loading. Such flexed hip postures are reached during vertical climbing in African apes (44). Repeated loading in these joint configurations is associated with increased trabecular reinforcement in the anterior subarticular region of the femoral head.

Together, these empirically grounded associations between joint posture, contact mechanics, and trabecular reinforcement in African apes provide the biomechanical basis for interpreting similar bone structural patterns in *P. robustus*. Dorsi- and plantarflexed ankle joints together with flexed hip and knee postures, characterize the mechanical conditions encountered during vertical climbing in African apes (44, 55), suggesting that *P*. *robustus* may have engaged frequently in such behaviors.

However, quantitative data on ankle, knee, and hip joint kinematics across different locomotor behaviors in wild great apes remain scarce reducing our ability to make direct comparisons with extant taxa. In addition, other postural and locomotor activities involving substantial lower limb flexion must be considered, particularly given that overlapping functional demands across different behaviors could result in similar trabecular distributions. For example, terrestrial quadrupedalism, including knuckle-walking, typically entails hip and knee flexion combined with ankle dorsiflexion. The comparatively closer trabecular pattern observed in *P*. *robustus* relative to gorillas could therefore be interpreted as reflecting similarities in habitual joint loading during terrestrial locomotion. However, terrestrial knuckle-walking does not involve sustained highly flexed hip postures or deep ankle plantarflexion, and therefore would not predict the same pattern of subarticular reinforcement (44, 54, 55). In addition, recent work has demonstrated that gorillas also engage extensively, more frequently than previously believed, in arboreal and vertical climbing (58). The possibility of a “bent-hip, bent-knee” (BHBK) terrestrial bipedal gait also warrants consideration. A BHBK configuration, characterized by sustained hip and knee flexion during bipedal locomotion, would alter loading orientations at the hip and knee compared to extended-limb bipedalism and could theoretically influence trabecular distribution. However, as in terrestrial quadrupedalism, such a gait would not involve flexed hip postures combined with substantial ankle plantarflexion, and therefore would not predict increased loading in the anterior region of the femoral head, and in the posterior region of the distal tibia, as observed in *P*. *robustus*, *Pan*, and *Gorilla* (59, 60). By contrast, squatting typically involves marked hip flexion and abduction, knee flexion, and ankle dorsiflexion, and could potentially generate a trabecular distribution similar to that documented here in the *P*. *robustus* femur and tibia (see discussion in ref. 23). Polished bone and horncore fragments recovered from *P*. *robustus* sites have been interpreted, based on experimental replication and use-wear analyses, as digging implements used to extract underground food resources (61–63), an activity that may have favored prolonged bouts of squatting. Nevertheless, experimental evidence indicates that static loading associated with sustained postures does not stimulate bone modeling or remodeling (64). Thus, only dynamic loading during squatting would be expected to produce a trabecular distribution comparable to that observed in the *P*. *robustus* lower limb.

Further insights into lower limb joint loading in both *P*. *robustus* and *A*. *africanus* and a strict one-to-one correspondence between trabecular structure and specific activities could be provided by examining trabecular bone orientation in addition to density (as measured here) and integrative biomechanical analyses. Currently, methodological constraints limit our ability to move beyond describing density distributions. The ongoing development of three-dimensional analyses of principal trabecular orientation (PTO) (65), micro-finite element analyses and inverse bone remodeling frameworks (66), additional kinematic information of wild African apes during different locomotor behaviors (67), and more biomechanically grounded experimental datasets—such as those derived from biplanar cineradiography based in vivo kinematics (68)—will allow more direct integration of joint motion, loading orientation, and internal bone architecture. These advances hold considerable promise for refining functional inferences and strengthening the theoretical links between morphology, mechanics, and behavior in fossil hominins.

Unlike *P*. *robustus*, *Australopithecus* specimens from Sterkfontein M4 do not show internal bone adaptations associated with high frequency of hip flexion and abduction, knee flexion, or high ankle plantar and dorsiflexion. The cortical and trabecular structures of the Sterkfontein M4 *Australopithecus* femur and tibia like those of modern humans, including a thick inferior cortex of the femoral neck, a single trabecular concentration in the posterior portion of the femoral head, and a high trabecular density in the anterocentral region of distal tibia articular surface. This evidence does not imply that Sterkfontein M4 *Australopithecus* was incapable of climbing (or squatting) (see, e.g., ref. 69), but that these behaviors were performed less frequently than in *P*. *robustus*. Indeed, the two Sterkfontein M4 *Australopithecus* distal tibiae exhibit differences in the observed rBV/TV distribution along the anterior margin, with StW 358 showing a greater reinforcement in this region relative to StW 389. This pattern potentially indicates that the StW 358 hominin experienced higher degree of ankle dorsiflexion than did the StW 389 hominin, as typically required during vertical climbing (38). The minimal differences in the external morphology of *P*. *robustus* and Sterkfontein M4 *Australopithecus* postcranial bones cannot account for the extent of the differences observed in their internal bone structures. Therefore, despite each having an unambiguous bipedal *bauplan*, *P*. *robustus,* and Sterkfontein M4 *Australopithecus* had different locomotor repertoires, at least in terms of the frequency or magnitude of particular behaviors. Given the relatively rapid turnover of trabecular tissue, the observed architecture likely reflects loading conditions experienced during at least the final months of life of the individuals investigated. While the investigated *P*. *robustus* individuals habitually combined terrestrial bipedalism with more varied positional behaviors requiring a flexed lower limb such as during vertical climbing, the present Sterkfontein M4 *Australopithecus* individuals habitually moved bipedally with an extended lower limb with less frequent flexed limb climbing. The external morphology of recently discovered upper limb elements confidently attributed to *Paranthropus boisei* (70–72), has also been interpreted as possibly functionally consistent with frequent use of arboreal locomotion. Future discoveries and analyses of the internal bone structure of the *P*. *robustus* (and *P*. *boisei*) upper limb will be important to further characterize the locomotor behavior of this southern African hominin species and how it compares to east African *Paranthropus*.

The scenario of differing locomotor repertoires in *P*. *robustus* and Sterkfontein M4 *Australopithecus* is consistent with the craniodental differences that have, since the 1950s, been interpreted as reflecting different dietary strategies in these taxa. A recent study of Plio-Pleistocene southern African bovid assemblages concluded that *P*. *robustus* was more likely to be a dietary generalist than was *A. africanus* (73). Moreover, among primates, generalist feeders tend to have smaller home ranges than specialist feeders (at an equivalent body mass) (74). Assuming this pattern holds true for early hominins, strontium isotopic studies indicate *P*. *robustus* had a smaller home range than did Sterkfontein M4 *Australopithecus* (75), further supporting the hypothesis that *P*. *robustus* is more of a generalist than Sterkfontein M4 *Australopithecus*. Accordingly, we propose that more generalized dietary habits and smaller home range in *P*. *robustus* may have elicited or selected for a greater diversity in locomotor behaviors compared with Sterkfontein M4 *Australopithecus*. In contrast, Sterkfontein M4 *Australopithecus* may have required more frequent terrestrial bipedalism to access a larger home range and obtain more specialized dietary resources. Altogether, the differences we reveal in locomotor repertoire, combined with craniodental morphology and dietary proxies, would be consistent with *P*. *robustus* exploiting a different ecological niche than Sterkfontein M4 *Australopithecus* within the same geographical region [and potentially for a short overlapping temporal period (5)], supporting the attribution of *P*. *robustus* to a distinct genus (14).

Our results show that the interaction of hominins with their environment is not always reflected in their external bony morphology (see ref. 76). The plasticity of internal bone structure to adapt to loading during life offers far greater insight into the complexity of the hominin locomotor repertoire beyond the single term “bipedalism.” The fact that the chronologically younger *P*. *robustus* shows a greater reliance on climbing than the older Sterkfontein M4 *Australopithecus* offers further evidence that the evolution of bipedalism (and behavior in general) was not linear within the hominin clade.

## Materials and Methods

### Extant Comparative Sample.

Our extant comparative sample (*SI Appendix*, Table S1) comprises only adult individuals from both females and males (or unknown sex) that had no visible signs of pathology. Our modern human (*H*. *sapiens*) sample derives primarily from individuals considered to have more sedentary lifestyles, including medieval individuals (Canterbury’s St. Gregory’s Priory, University of Kent, UK), early 20th century anatomical collections (Georg-August-Universität Göttingen, Germany), and more recent collections (Pretoria Bone Collection, Pretoria, southern Africa; W.M. Bass Collection, University of Tennessee, USA). Whenever possible individuals aged between 21 and 50 y old have been selected. For the study of femoral neck cortical thickness, two females aged 50 y old from McGregor Museum, Kimberley, southern Africa, are from a hunter-gatherer population (*SI Appendix*, Table S1; for the modern human comparative sample, ethical clearance was obtained from the Faculty of Health Sciences Research Ethics committee of the University of Pretoria, ref. no. 39/2016). The *P*. *troglodytes* sample is from a wild, habituated population within the Taï National Park, Côte d’Ivoire, often of known sex and age, curated through the Taï Chimpanzee Project at the Max Planck Institute for Evolutionary Anthropology, Leipzig, Germany. This population lives within a dense tropical forest with an overall arboreality of 48.9% for females and 64.8% for males (77). The *G*. *gorilla* sample also derives from wild-caught individuals, primarily from Cameroon, curated at the Powel Cotton Museum (Birchington on Sea, UK). Although the locomotion of these particular individuals is not known, *G*. *gorilla* are among the most arboreal of the gorilla species, with populations in Gabon spending between 19% (adult males) and 34% (adult females) of their time in the trees (58).

### Scanning Procedure, Orientation, Virtual Extraction, and Segmentation.

*SI Appendix*, Tables S1 and S2 provide details on the different microtomographic (microCT) equipment and the resulting scan resolutions for the fossil and extant specimens, respectively. Prior to analysis, all specimens were reoriented to standardized anatomical positions and cropped using Avizo 9.0 (FEI Visualization Sciences Group). The femoral neck was demarcated medially by the head–neck junction and laterally by the superior flaring of the greater trochanter. This definition follows the method developed by Ruff and Higgins (78) and used by Profico et al. (46) demonstrated that intra- and interobserver variations in the orientation of the femoral neck and potential delimitations of the femoral neck do not influence results of cortical thickness distributions. All extant and some fossil proximal and distal femora (SK 82, SK 97, StW 318, StW 479, StW 522) were first segmented using the MIA-Clustering segmentation (79) to automatically isolate the bone. For the fossils StW 358, StW 389, SWT1/HR-2c, and SWT1/LB-2, the Trainable Weka Segmentation plugin available in Fiji (80) was used as it allows more accurate separation of trabecular bone from surrounding inclusions in these particular specimens. Finally, SWT1/HR-2b is the most poorly preserved distal femur with substantial matrix surrounding the thin trabeculae (*SI Appendix*, Fig. S2). Therefore, for this specimen, a deep learning approach to segmentation of the bone was used (81); the U-Net 2.5 model of 7 slices has been trained and applied to the fossil in Dragonfly 2024.1 [(Computer software) Comet Technologies Canada Inc., Montreal, Canada; software available at https://dragonfly.comet.tech/] using 100 epochs, 5 training frames, a 0.25 stride ratio and 256 batch size. For the distal femur, to ensure that differences in segmentation methods between the Sterkfontein M4 *Australopithecus* specimen (StW 318) segmented using Mia-Clustering segmentation and the *Paranthropus* specimen (SWT1/HR-2b) segmented with Dragonfly did not substantially affect the results, we resegmented the Sterkfontein M4 *Australopithecus* distal femur (StW 318) using the same Dragonfly deep learning model applied to the other specimen and compared these results to those obtained using the MIA-clustering segmentation. The results indicate that the differences introduced by the two segmentation procedures are minimal: only 3 out of 990 pairwise comparisons show a Euclidean distance (based on the first three PCs) lower than the distance observed between the two independent segmentations of specimen StW 318. (*SI Appendix*, Fig. S14)

### Methods for the Trabecular Bone Analysis of the Distal Tibia, Distal Femur, and Femoral Head.

Cortical and trabecular bone were then separated in the segmented images in medtool 4.7 (https://www.dr-pahr.at/medtool/). By projecting a series of rays across the scan, and applying morphological filters, medtool 4.7 assigns unique scalars to the trabecular, cortical, internal air, and background. The trabecular and cortical bone regions were then separated to analyze the trabecular tissue independently (82, 83).

The canonical holistic morphometric analysis (cHMA) protocol uses a statistical deformation model to create, for each investigated anatomical site, a canonical shape that can be morphed to all the bones in each sample in 3D space (33). Two iterations of a similarity and b-spline registration were run on all bones of the extant sample. The registration process results in a solid canonical distal tibia, distal femur, and femoral head trabecular volume, representing the mean size, shape, and position of the internal trabecular bone space of the entire sample (i.e., all extant taxa) for each element (33). The canonical model was then meshed and morphed to individual trabecular volumes using the inverse of transformation data from the statistical deformation model. Holistic morphometric analysis (HMA) was then run in medtool 4.7 to measure bone volume fraction (BV/TV) (82, 83). A 3D grid with nodes spaced 2.5 mm apart was placed over each individual trabecular volume. A 5 mm sampling sphere was centered on the nodes measuring trabecular BV/TV. These BV/TV values were interpolated onto individual meshes that could then be morphed back to the canonical shape while retaining individual BV/TV distributions. Color maps representing the distribution of BV/TV were viewed in Paraview v.5.12.0. Since data were collected on the canonical mesh, the tetrahedra of this mesh are geometrically homologous among individuals and among taxa for each anatomical region.

The medial portion of the distal tibia excluding the malleolus, the lower region of the lateral condyle of the distal femur, and the superior portion of the femoral head were virtually extracted on the canonical meshes using Paraview v.5.12.0, such that only the mesh elements preserved in all fossil specimens were analyzed. More precisely, on the canonical mesh of the distal tibia, a sagittal cutting plane was positioned at the level of the extreme medial edges of the anterior and posterior margins, thereby excluding the entire malleolus at the border of the medial malleolar surface. On the canonical mesh of the distal femur, a plane parallel to the shaft axis was positioned at the center of the intercondylar fossa, separating the mesh into two regions (the lateral and the medial) and was used as a reference to extract the lateral region for analysis. The inferior region of the lateral condyle was defined as the portion distal to a coronal plane passing through the most posterior point of the condyle. Everything distal (below) this plane was kept for analysis. On the canonical mesh of the femoral head, a plane parallel to the main axis of the neck and positioned at the level of the center of the fovea capitis—thus separating the head into superior and inferior portions—was used as a reference to extract only the superior portion for the analyses. Finally, for each of the three anatomical elements, on the selected portions (i.e., the distal tibia excluding the malleolus, the lateral condyle of the distal femur and the superior region of the femoral head), the BV/TV of each tetrahedron was divided by the average BV/TV of all tetrahedra in each individual to produce a dataset of relativized values of BV/TV or rBV/TV. While absolute bone BV/TV is informative, rBV/TV aims to control for systemic differences in trabecular structure across individual and particularly across taxa (e.g., ref. 84). rBV/TV and the spatial distribution of rBV/TV in the mesh demonstrates where bone volume has increased or decreased relative to the mean of that bone. We assume that regions of higher rBV/TV reflect higher habitual loading (e.g., refs. 34 and 35).

### Methods for the Cortical Thickness Analysis of the Femoral Neck.

By using RStudio v.1.2.5033 running with R v. 3.4.4 (R Core Team, 2018) and specifically the package morphomap (46) on each 3D surface model, we extracted 100 cross sections along the extracted femoral neck defined at equally spaced increments of 1% between 0 (medial cross section) and 100% (lateral cross section) of the neck length. Following the methodological steps outlined in (46) by using a set of 180 equiangular semilandmark pairs for each individual, a matrix (by default, 180 pairs of semilandmarks by 100 slices) of cortical thicknesses was created. The absolute values (i.e., the observed cortical bone thickness) were smoothed using a generalized additive model within the package mgcv in R (85). A smoothing step is essential to avoid comparing microvariations between individuals and taxa, only taking into consideration the general pattern of bone thickness topographic distribution along the neck. To compare distribution patterns without considering taxon-specific variation in terms of magnitude of the absolute values of cortical thickness, which might relate to variation in body mass between individuals, we standardized each smoothed cortical thickness value by the mean thickness of that entire neck (i.e., relative mean smooth cortical bone thickness; rmsCBT) for each individual. Unrolled cortical thickness maps represent the 180 pairs of semilandmarks in the x-axis starting from the most anterior pair, and in the y-axis the 100 cross sections starting with the most medial section. These were created using ggplot package in R (86).

### Statistical Analyses of the Results of Relative Bone Volume Fraction and Cortical Thickness.

For each skeletal element and for the trabecular and cortical analyses, we conducted a PCA in RStudio v.1.2.5033 running with R v. 3.4.4 (R Core Team, 2018) using the stats package (v. 3.6.2). For trabecular analysis, rBV/TV values of each tetrahedron in each of the three canonical meshes were used as input variables. For cortical analysis of the femoral neck, rmsCBT values between each pair of landmarks were used as input variables. To test if overall rBV/TV and rmsCBT distributions were significantly different between extant taxa, a permutational MANOVA was run on the Euclidean distance matrix that described the first three PC scores of each dataset (as they explain for more than 50% of the cumulative variance), using the Vegan packages in R (87). Before these tests were conducted, a test of multivariate homogeneity of variance was performed on the Euclidean distance matrix that describes the PC scores and a Bonferroni correction was applied to all pairwise results to ensure valid comparisons. As not all data met the assumption of multivariate normality, a permutation approach was taken. For all statistical tests, a *P*-value of < 0.05 was considered significant. We also conducted cross-validated canonical variates analyses (CVAs) using the extant groups presented in *SI Appendix*, Table S2 using the R package Morpho (88). Since CVA computation requires the number of variables to be much smaller than the number of specimens, we computed the CVA based on a subset of the first PC scores showing the highest degree of correct classification [screening the correct classification results and selecting the minimum number of PC scores enabling to reach the optimum of correct classification (89)]. The group affinities of the fossils were assessed by projecting them into CVAs before calculating typicality probabilities.

## Supplementary Material

Appendix 01 (PDF)

## Data Availability

The microCT scans of fossil and extant specimens are available in the Human Fossil Record Archive (https://human-fossil-record.org) (90) except for the microCT scans of the human femora from the Pretoria Bone Collection at the Department of Anatomy of the University of Pretoria that are available on the Digital Repository of the *Bakeng se Afrika* program (contact detail: FARC@up.ac.za).
