# Supplementary material for: Swartkrans Paranthropus and Sterkfontein Australopithecus from southern Africa had different locomotor repertoires
Source: Proc Natl Acad Sci U S A. 2026 May 11;123(20):e2532193123. doi: 10.1073/pnas.2532193123 (PMC13187710; doi:10.1073/pnas.2532193123)
Supplement: Supplementary file 1 — Appendix 01 (PDF) [file pnas.2532193123.sapp.pdf]

PNAS

Supporting Information for
Swartkrans *Paranthropus* and Sterkfontein *Australopithecus* from southern Africa had different locomotor repertoires

Marine Cazenave[1,2,3]*, Annalisa Pietrobelli[1], Andrea Luková[1,4], Sebastian Bachmann[5], Matthew V. Caruana[6,7], Ronald J. Clarke[6], Christopher J. Dunmore[8], Ashley S. Hammond[9,10,2], Jason L. Heaton[6,11], A. J. Heile[12], Jakobus Hoffman[13], Kathleen Kuman[14], Dieter H. Pahr[5], Christopher M. Smith[15,2,16], Dominic Stratford[14,17], Alexander Synek[5], Zewdi J. Tsegai[18], Tracy L. Kivell[1], Travis Rayne Pickering[12,6], Matthew M. Skinner[19]

Affiliations:
[1]Department of Human Origins, Max Planck Institute for Evolutionary Anthropology, Leipzig, Germany
[2]Division of Anthropology, American Museum of Natural History, New York, USA
[3]Department of Anatomy, Faculty of Health Sciences, University of Pretoria, Pretoria, South Africa
[4]Department of Anthropology, University of West Bohemia in Pilsen, Pilsen, Czech Republic
[5]Institute of Lightweight Design and Structural Biomechanics, TU Wien, Vienna, Austria.
[6]Evolutionary Studies Institute, University of the Witwatersrand, Johannesburg, South Africa
[7]Palaeo-Research Institute, University of Johannesburg, Johannesburg, Auckland Park 2006, South Africa
[8]School of Biosciences, University of Kent, Canterbury, UK
[9]Institut Català de Paleontologia Miquel Crusafont (ICP-CERCA), Universitat Autònoma de Barcelona, Barcelona, Spain
[10]Institució Catalana de Recerca i Estudis Avançats (ICREA), Barcelona, Spain
[11]Department of Biology, University of Alabama at Birmingham, Birmingham, Alabama, 35205, USA
[12]Department of Anthropology, University of Wisconsin-Madison, Madison, Wisconsin, USA
[13]South African Nuclear Energy Corporation, Pelindaba, South Africa
[14]School of Geography, Archaeology and Environmental Studies, University of the Witwatersrand, Johannesburg, South Africa
[15]Department of Biology, Fairfield University, Fairfield, CT, USA
[16]New York Consortium in Evolutionary Primatology, New York, NY, USA
[17]Department of Anthropology, Stony Brook University, Stony Brook, USA
[18]Department of Organismal Biology and Anatomy, University of Chicago, Chicago, USA
[19]Department of Archaeogenetics, Max Planck Institute for Evolutionary Anthropology, Leipzig, Germany

*Corresponding author: Marine Cazenave
Email: marine_cazenave@eva.mpg.de

This PDF file includes:

Supporting text
Figures S1 to S14
Tables S1 to 3
SI Appendix, II
SI References

Supporting Information Text

Text S1: Previous cortical and trabecular analyses of *A. africanus* and *P. robustus* postcranial remains

Although many uncertainties persist regarding the functional relationship between the external morphology and the internal cortical and trabecular bone structure, experimental evidence has demonstrated that internal bone tissues can offer insight into site-specific loading conditions experienced throughout an individual's life (1–15). Therefore, in the last three decades, an increasing number of studies have comparatively investigated variation in internal bone structure to draw functional inferences [e.g., (16-22); see (23) for references before 2016], including in the southern African australopiths (see details in (24)).

In the distal tibia, previous studies of trabecular bone density and the degree of anisotropy (a measure of alignment among trabecular struts) show that differences in trabecular structure in humans and *Pan* are consistent with differences in habitual dorsiflexion at the ankle (25–28). Only one study has investigated trabecular structure of the distal tibia of fossil hominins, focusing on "*A. africanus*", and applying a volume-of-interest approach (25). This study showed that the trabeculae are highly aligned and oriented in a similar manner to that of humans and distinct from *Pan* (25). Indeed, the trabecular bone orientation of the distal tibia implies the use of talocrural joint plantarflexion in *A. africanus*, which modern humans use at the end of the push-off phase, a distinctive feature of obligate bipedalism (25,29,30). Now with the recent discovery of the first *P. robustus* distal tibia from Swartkrans Member 1 (31), we are conducting the first analysis of the trabecular organization of the *P. robustus* distal tibia.

In the distal femur, previous studies of extant primates have identified differences in the distribution of trabecular bone volume fraction (BV/TV) that are consistent with locomotor differences. In particular, humans show a distinct pattern compared with great apes that reflects the loading conditions of knee extension during bipedal locomotion (21,32,33). To date, no analyses of trabecular structure of *Australopithecus* and *Paranthropus* distal femora have been conducted. The recent discovery of the first distal femur of *P. robustus* offers the unique opportunity to quantify the pattern of trabecular structure in this taxon.

In the femoral head, previous analyses have shown differences in the trabecular structure across primate taxa (i.e., strepsirrhines to hominoids) displaying different locomotor behaviors (34–45). However, most of these studies have applied a volume-of-interest (VOI) approach, quantifying trabecular structure in only a subregion of the femoral head. A VOI approach was also used to investigate the trabecular structure of the *Australopithecus* femoral head, showing a pattern more similar to that of modern humans and Pleistocene *Homo* than to the typical extant ape condition [see discussion for possible differences that require further investigation of trabecular distribution of the femoral head of *P. robustus* in (20)]. A more recent analysis using a whole-epiphysis approach revealed that extant non-human apes show two concentrations of high BV/TV (anterior and posterior) within the femoral head, while modern humans only exhibit a posterior bone concentration, consistent with their respective differences in habitual hip postures and loading (45). A subsequent study identified that an *Australopithecus* specimen from Sterkfrontein Member 4 shows a pattern that resembles that of humans while another Sterkfontein specimen from Member 5 East shows a more ape-like pattern. However, the taxonomic attribution of the latter specimen is uncertain (46). *P. robustus* specimens from Swartkrans Member 1 have not been previously investigated with a whole-epiphysis approach.

In the femoral neck, the superoinferior asymmetry in cortical bone thickness has been well studied. Modern humans are characterized by a distinctly asymmetric distribution resulting from an absolutely thinner superior cortex and a thicker inferior cortex related to the distinct loading conditions of obligate, upright bipedal locomotion (e.g. (47)). Conversely, non-human great apes possess a more symmetrical distribution of cortical bone across the femoral neck (48,49) reflecting more variable loading of the hip joint, and particularly hip abduction and flexion, during a diversity of locomotor behaviors (50). In 2D analyses, it has been shown that australopiths (including southern African *Australopithecus* and *Paranthropus*) have relatively more symmetric superior and inferior femoral neck cortices at the mid-neck than modern humans, but a more human-like pattern at the base of the neck, suggesting greater superior loads at midneck during bipedal locomotion than in humans (47,51). A more recent analysis of *P. robustus* femora, investigating the

entire femoral neck in 3D, has revealed a less human-like pattern in the internal cortical bone structure than previously thought, suggesting that the locomotor loading of the hip joint may have differed from later hominins (16). However, it remains unknown how *Australopithecus* femoral specimens compare and if they show a similar cortical bone pattern to that of *P. robustus*.

Text S2: Results of the trabecular bone distribution of the distal tibia in *Australopithecus*, *P. robustus*, modern humans and African apes

In our principal component analysis (PCA; Fig. 1 and *SI Appendix,* Fig. S3), the first three principal components of trabecular bone distribution (PCs) collectively explain 50.8% of variance (PC1: 26.8%; PC2: 14.9%; PC3: 9.1%). Modern humans plot towards PC1 negative scores while African apes plot toward PC1 positive scores (*SI Appendix,* Fig. S3). *Gorilla* and *Pan* are separated along PC2, with *Pan* plotting towards PC2 positive scores. The bivariate plot of PC1-PC3 shows a separation between modern humans and African apes across PC1.

The PC1 negative extreme, characterizing modern humans, reflects high rBV/TV inferiorly at the centre of the tibiotalar subarticular surface. Similarly, in midsagittal view, the area of high rBV/TV is concentrated at the centre of the tibiotalar subarticular surface. Indeed, the tetrahedra driving the loadings (i.e., those above 22th percentile) along PC1 negative scores are located at the central-medial area of tibiotalar subarticular surface. Conversely, the PC1 positive extreme, characterizing African apes, reflects high rBV/TV along the anterior margin and posterocentrally on the posterior margin of the tibiotalar subarticular surface. In midsagittal view, the anterior and posterior concentrations of trabecular bone are also marked, not extending to the subarticular centre but expanding proximally at the anterior margin. The tetrahedra driving the loadings along PC1 positive scores are located on the anterolateral, anteromedial and posterior sections of the subarticular surface.

The PC2 and PC3 negative extremes, characterizing modern humans, reflect high rBV/TV inferiorly at the centre of the tibiotalar subarticular surface. Compared to PC1 negative extreme, the area of high rBV/TV is more expanded towards the subarticular margins. However, as seen in midsagittal view, the area of high rBV/TV remains confined at the centre of the tibiotalar subarticular surface and does not extend proximally towards the anterior margin. Similar to PC1 negative extreme, the tetrahedra driving the loadings along PC2 negative scores are located at the central-medial area of tibiotalar subarticular surface, while the tetrahedra driving the loadings along PC3 negative scores are located laterally on the tibiotalar subarticular surface. The PC2 positive extreme characterizing *Pan* reflects high rBV/TV along the anterolateral, anteromedial and posterocentral regions of the subarticular surface. In midsagittal view, the anterior and posterior concentrations of trabecular bone are also marked, not extending to the subarticular centre but expanding proximally at the anterior and posterior margins. The tetrahedra driving the loadings along PC2 positive scores are located on the anterior and posterior margins of the fibular incisura, laterally on the tibiotalar subarticular surface. The PC3 positive extreme, characterizing modern humans and *Gorilla*, reflects high rBV/TV along the anterior margin and posterocentral section of the subarticular surface. In midsagittal view, the anterior and posterior concentrations of trabecular bone are also marked, slightly extending towards the subarticular centre from the posterior margin but expanding proximally at the anterior margin. The tetrahedra driving the loadings along PC3 positive scores are located centro-laterally on the tibiotalar subarticular surface.

The 3D PCA plot (*SI Appendix,* Fig. S3) confirms the previous observations of both fossil and extant samples. Modern humans cluster separately, while *Pan* and *Gorilla* slightly overlap. *P. robustus* plots within the cluster of *Gorilla* on both PC1-PC2 and PC1-PC3 bivariate plots, while the two *Australopithecus* individuals plot in their own morphospace. In the PC1-PC3 bivariate plot, StW 389 plots within the modern human cluster while StW 358 plots in its own morphospace close to the modern human cluster.

Multivariate statistics calculated with pairwise PerMANOVA on the Euclidean distance matrices of the combined PC scores along the first three PC axes (as explaining higher than 50% of the cumulative variance) reveal a significant difference among all extant taxa (*SI Appendix*, Table S3).

The Euclidean distances between the *Paranthropus* and *Australopithecus* specimens, calculated from the first three principal components, exceed the upper 5% of within-group distances in all three extant taxa. The only exception is the distance between StW 358 and SWT1/HR-2c, which exceeds 65% of within-group distances in modern humans and 86% in gorillas (*SI Appendix*, Fig. S3).

CVA (*SI Appendix,* Fig. S4) was conducted using the first five PCs (see Methods section about decision on number of PCs included), which collectively explain 62.54% of the total variance. The first two canonical variates (CV1 and CV2) explain 90.04% and 9.66% of the variance in the canonical space, respectively. *P. robustus* plots within the *Gorilla* distribution, while the two *Australopithecus* specimens plot variably in their own morphospace, in between modern humans and African apes. Typicality probabilities reveal that StW 358 is best assigned to *Pan* (9.4% probability), StW 389 is not assigned to any group, and that SWT1/HR-2c is assigned to *Gorilla* (9.6% probability).

Text S3: Differences in the *Australopithecus* sample of the distal tibia

The results of the study revealed variability in the trabecular distribution of the distal tibia between the fossil specimens assigned to *Australopithecus* from Sterkfontein Member 4. Both StW 358 and StW 389 share a concentration of bone density localised at the antero-central part of the subarticular surface. This result is consistent with previous volume-of-interest analyses showing a modern human-orientation of the trabeculae in Sterkfontein tibiae (25), compatible with an extended lower limb posture. This interpretation is also supported by experimental analyses of contact point at tibiotalar surface in modern humans during load-bearing, documenting an anterocentral location of average contact area with the talar trochlea and region of maximum pressure (52–54).

The differences in the PCA scores between StW 358 and StW 389 can be explained by differences in the locations of high rBV/TV concentrations. Both *Australopithecus* specimens show a region of high rBV/TV along the anterior margin of distal tibial articular surface, running mediolaterally throughout the whole anterior aspect in StW 358 and more centrally confined in StW 389. However, in midsagittal view, StW 389 displays a region of high rBV/TV that is concentrated at the centre of the tibiotalar subarticular surface and expands anteriorly but does not reach the margin and does not expand proximally. Conversely, StW 358 shows a more marked concentration of trabecular bone anteriorly compared to StW 389, which also expands proximally at the anterior margin, resembling that of great apes, and is likely linked to loading in dorsiflexion (25,55). Moreover, StW 389 shows an overall lower rBV/TV compared to StW 358.

Both StW 358 and StW 389 are traditionally attributed to *A. africanus*, but at least two hominin ‘morphs’ (56,57) or species (58) derived from Member 4 deposits at Sterkfontein based on substantial craniodental and postcranial morphological variation (56,57,58). Despite past and ongoing efforts to clarify the location and morphology of the boundary between M4 and M5 and other stratigraphic complexities at Sterkfontein significant challenges remain in distinguishing the deposits of Member 4 and Member 5 near their decalcified and irregular boundary in the centre of the surface site (59–63).

Text S4: Results of the trabecular bone distribution of the distal femur in *Australopithecus*, *P. robustus*, modern humans and African apes

In a recent analysis, Lukova et al. (32) investigated the rBV/TV distribution in the distal femoral epiphysis. Modern humans show concentrated high rBV/TV in the posteroinferior regions of both femoral condyles, especially the lateral condyle, and at cruciate ligament insertion points, while African apes display more homogeneous and deeper rBV/TV patterns, particularly in *Pan* compared to *Gorilla*, with notable variation in condylar and ligament insertion regions. The present results quantifying just the preserved trabeculae in the lateral condyle are in line with these previous observations (32). PC1, which explains 25.9% of the variance, separates modern (negative PC1) from *Pan* (positive PC1) while *Gorilla* is intermediate between the two taxa (Fig. 2 and *SI Appendix,* Fig. S5). SWT1/HR-2b plots within the *Pan* range of variation and StW 318 plots between the modern and *Pan* distribution, but closer to the modern distribution. Positive PC1 scores are associated with higher rBV/TV values in the posteroinferior regions of the lateral condyle and under the lateral epicondyle, whereas negative PC1 scores are associated with higher rBV/TV values under the patellar surface.

PC2 accounted for 14.5% of the remaining variation in rBV/TV values and separates *Gorilla* from modern humans and *Pan*. SWT1/HR-2b plots within the *Pan* distribution and StW 318 plots with modern humans and *Pan* on the negative side of PC2. Positive PC2 scores distinguish *Gorilla* with a more anterior concentration of rBV/TV under the patellar surface compared to higher posterior rBV/TV for negative PC2. Moreover, positive PC2 is associated with higher rBV/TV values in the posteroinferior region of the lateral

condyle and under the lateral epicondyle, which distinguishes *Pan* and modern humans (Fig. 2 and *SI Appendix,* Fig. S5).

The main difference observed between the two fossil specimens is the modern human-like distribution of rBV/TV on the patellar surface in StW 318 while SWT1/HR-2b shows a more African-ape like distribution, suggesting that the knee of *P. robustus* might have experienced higher (or more frequent) knee flexion (Fig. 2).

The more anterior rBV/TV concentration on the patellar surface of the femur found in SWT1/HR-2b, which causes it to fall closer to the *Gorilla* distribution on PC2, may be explained by several biomechanical factors:

1. The anterior side of the patellar surface is subjected to substantial compressive forces during knee flexion and extension. These forces are especially pronounced when the knee is in a flexed position. Thus SWT1/HR-2b might have flexed their knees more compared to StW 318 specimen.

2. To ensure stabilization pf the knee, muscles may have been contracted more strongly, creating greater internal forces at the contact area of the patella.

3. The anterior patellar surface experiences higher loading in greater knee flexion, which might have been more common in SWT1/HR-2b compared to StW 318.

The statistical analyses confirmed these observations. The multivariate pairwise comparisons (PerMANOVA) calculated on the Euclidean distances of combined PC scores along the first three PC axes (49.5% of cumulative variance explained) reveal a significant difference among all extant taxa (*SI Appendix*, Table S3).

The Euclidean distances between the *Paranthropus* and *Australopithecus* specimens, calculated from the first three principal components, exceed the upper 5% of within-group distances in *Pan* and *Gorilla*, and the upper 13% of the modern human group (*SI Appendix*, Fig. S5).

In addition, a CVA (*SI Appendix,* Fig. S6) was conducted using the first two components that represent the lowest number of components that explain the highest variance. The first two canonical variates (CV1 and CV2) explain 62.35% and 37.65% of the variance in the canonical space, respectively. *P. robustus* plots within the cluster of *Pan*, while *Australopithecus* plots in its own morphospace between modern humans and African apes. Typicality probabilities assign the StW 318 to modern humans (13% probability) and SWT1/HR-2b to *Pan* (51% probability).

Text S5: Results of the trabecular bone distribution of the femoral head in *Australopithecus*, *P. robustus*, modern humans and African apes

Two previous studies investigated variation in the trabecular distribution patterns within the femoral head in great apes and modern humans by using similar holistic methods employed here (i.e., using medtool software for a whole bone analysis) (45,46). Qualitative distribution and quantitative comparisons of rBV/TV values showed that trabecular bone distribution patterns reflected differences in extant ape locomotor patterns. *Pan* and *Gorilla* displayed two regions of high rBV/TV within the femoral head: one in the posterior and one in the anterior aspect of the femoral head, consistent with frequent used of both flexed and extended hip postures during terrestrial quadrupedalism and vertical climbing, the modes of locomotion that comprise the majority of *Pan* and *Gorilla* locomotion (64–66). However, unlike *Pan*, these regions were better defined and more discrete in most *Gorilla* individuals (11 of 14 individuals) (45,46). This more discrete pattern is perhaps due to their greater body mass, which has been shown to restrict range of motion at some joints (67) and may explain the more well-defined concentrations in *Gorilla*. The two concentrations are closer to each other in *Gorilla* than in *Pan*, which is also consistent with the reduced range of motion at the hip joint of *Gorilla* (67,68). However, these previous studies of femoral head trabecular bone did not map and quantify the trabecular patterning to a canonical mesh, thus limiting homologous statistical comparisons.

Our results confirm previous observations. By interpolating individual distributions of rBV/TV onto the canonical mesh, we observe that five out of 10 *Pan* individuals show two distinct concentrations of high rBV/TV, while the five remaining individuals show less discrete concentrations, with connected anterior and posterior regions of high rBV/TV concentrations (*SI Appendix,* Fig S7). For our *Gorilla* sample, all individuals display two distinct regions of high rBV/TV concentration. Our results also confirmed that modern humans display one main region of high rBV/TV, located posteriorly and superiorly on the femoral head. This

concentration was positioned more medially than the posterior concentration seen in great apes and closer to the fovea capitis, which is consistent with loading of the femur at a valgus angle (*SI Appendix,* Fig S7) (45) during bipedalism.

The statistical analyses confirmed these results (Fig. 3 and *SI Appendix,* Figs. S7 and S8). PC1 explains 30.4% of the total variance and distinguishes humans, with one rBV/TV concentration (positive PC1 scores) from African apes with two rBV/TV concentrations (negative PC1 scores). PC2 explains 14.7% of the remaining variance and the extreme configurations reflect differences in the position of the rBV/TV concentrations but do not distinguish well among our study taxa (*SI Appendix,* Fig. S8). The pairwise perMANOVA tests run on the first three principal components confirmed that modern humans are distinguished from both African ape taxa, while *Pan* and *Gorilla* are not significantly different from each other (SI Appendix Table S3). The Euclidean distances between the *Paranthropus* and *Australopithecus* specimens, calculated from the first three principal components, exceed the upper 16% of within-group distances in *Pan*, 28% in *Gorilla*, and 48% the modern human group (*SI Appendix*, Fig. S8).

The CVA results based on the first five components that represent the lowest number of components that explain the highest variance confirming that the groups observed are not spurious. The results of the CVA support the previous observations of discrimination between the taxa in the PCA results (*SI Appendix,* Fig. S9). Typicality probabilities assign the *Australopithecus* specimen to humans (30% probability) and the *P. robustus* to *Gorilla* (50% probability) (*SI Appendix,* Fig. S9).

Text S6: Result of the cortical bone distribution of the femoral neck in *Australopithecus*, *P. robustus*, modern humans and African apes

Cortical bone distribution at the femoral neck has been shown to reflect biomechanical loading during locomotion (48–51,69,70). Within extant and extinct hominids, differences in superoinferior asymmetry of cortical thickness at the femoral neck have been ascertained and interpreted as related to posture/locomotion (16,47–49,51,69,71). In a previous study (16), morphometric maps extended the comparative analysis to the entire femoral neck including the anterior and posterior aspects, showing that modern humans and *Pan* differ in the global pattern of cortical bone distribution. For the first time here, we statistically investigated the differences in the cortical thickness distribution along the entire femoral neck of modern humans, *Pan* and *Gorilla*. For each taxa, the mean ‘unrolled’ maps of the smoothed cortical bone thickness variation (standardised by the mean thickness projected in a matrix of 127 pairs of semi-landmarks (x-axis) of 100 slices (y-axis) extracted along the femoral neck) are presented in SI Appendix Fig. S10, along with the 3D cortical thickness neck variation projected onto a representative individual of the taxa. The main differences between humans and great apes are found in the superior portion of the lateral half of the neck, which is the thinnest region of the human neck but much thicker in African apes (*SI Appendix,* Figs. S10, S11). There is a general trend of lateral to medial decrease of the cortical thickness in all taxa, resulting in a less heterogenous cortical distribution between the four quadrants in the medial portion of the neck.

The PCA (*SI Appendix*, Fig. S11) as well as the cross-validated CVA (*SI Appendix*, Fig. S12) of the first five PCs significantly (*SI Appendix*, Table S3) discriminate modern humans and African great apes as well as *Pan* from *Gorilla*. The PCAs of cortical bone thickness distribution across the femoral neck show that both *Australopithecus* specimens fall in their own morphospace, but fall closer to the modern human distribution (*SI Appendix*, Fig. S11). *P. robustus* falls closer to the African great ape range of variation in the first three components explaining for 71.2% of the variance (*SI Appendix*, Fig. S10, S11). The results of the CVA support the previous observations of discrimination among the taxa in the PCA results (*SI Appendix*, Fig. S12). Typicality probabilities assign both *Australopithecus* specimens to modern humans (51% for StW 426 and and 20% for StW 469) while both *P. robustus* specimens are assigned to *Gorilla* (40% for SK 29 and 16% for SK 44) (*SI Appendix*, Fig. S12).

The Euclidean distances between StW 479 and the two *Paranthropus* specimens calculated from the first three principal components, exceed the upper 5% of within-group distances in all three extant taxa. The Euclidean distances between StW 522 and the two *Paranthropus* specimens calculated from the first three principal components, exceed the upper 18% of within-group distances in all three extant taxa and even exceeding 1% for the within-group distances in gorilla (*SI Appendix*, Fig. S11).

As shown by the unrolled cartographies of the smoothed cortical bone thickness variation standardised by the mean thickness, both *P. robustus* SK 82 and SK 97 specimens are distinguished from *Australopithecus* in having well-developed cortical bone reinforcement at the superior aspect of the neck,

while this reinforcement is absent in StW 479 and StW 522 (*SI Appendix*, Fig. S10). As the anterior reinforcement identified in *P*. *robustus* is not present in any individuals in our extant hominid comparative samples or in *Australopithecus*, we consider it to be a feature potentially unique to *P*. *robustus*. As observed for the extant taxa, such differences between fossil specimens are constrained to the lateral portion of the neck as they also show a general trend of lateral to medial decrease of the cortical thickness resulting in a less heterogenous cortical distribution between the four directions in the medial portion of the neck (*SI Appendix*, Fig. S10).

Figures

Fig. S1. 3D rendering of the hominin femora and tibiae investigated in this study and external morphological features associated with ankle, knee and hip joints in southern African *Paranthropus* and *Australopithecus*. Morphological information on the proximal femur is from (72-78), on the knee from (74,75, 79-82) and on the distal tibia from (74,75,83-85).

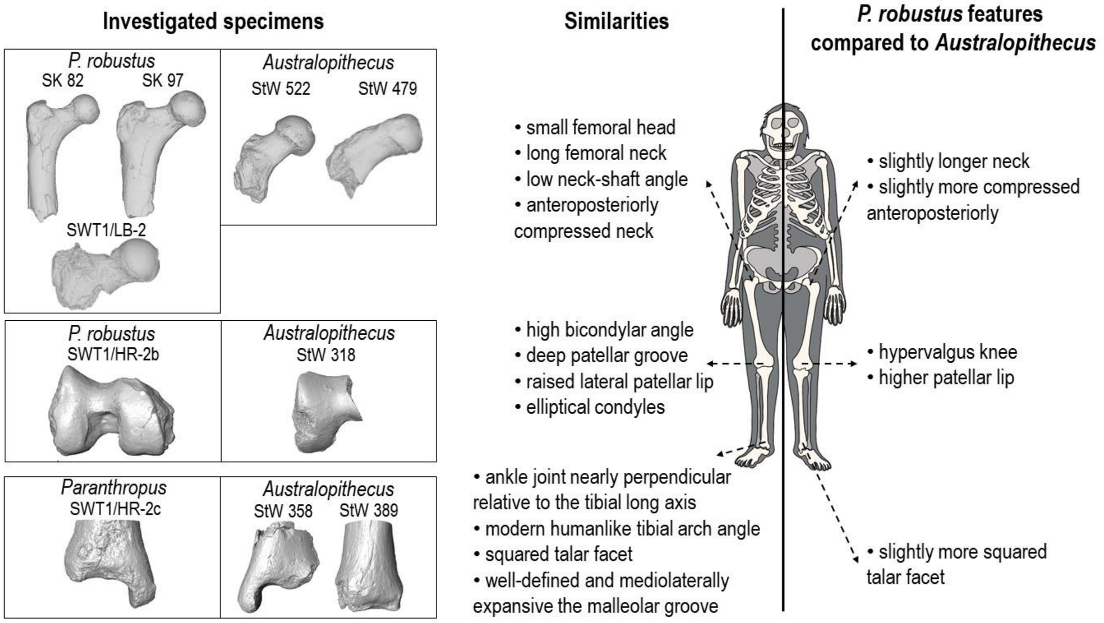

Fig. S2. Preservation of the internal bone structure of all the fossils included in the study. (A) *P. robustus* SWT1/HR-2c and *Australopithecus* StW 358 and StW 389 distal tibiae; (B) *P. robustus* SWT1/HR-2b and *Australopithecus* StW 318 distal femora; (C) *P. robustus* SK 82, SK 97 and SWT1/LB-2 proximal femora and *Australopithecus* StW 479 and StW 522 proximal femora. For each element, the top row depicts a 3D rendering of the external morphology; middle row shows a central cross-section of raw microCT data extracted within the plane of view; bottom row shows the segmented cortical (femoral neck only) and trabecular bone.

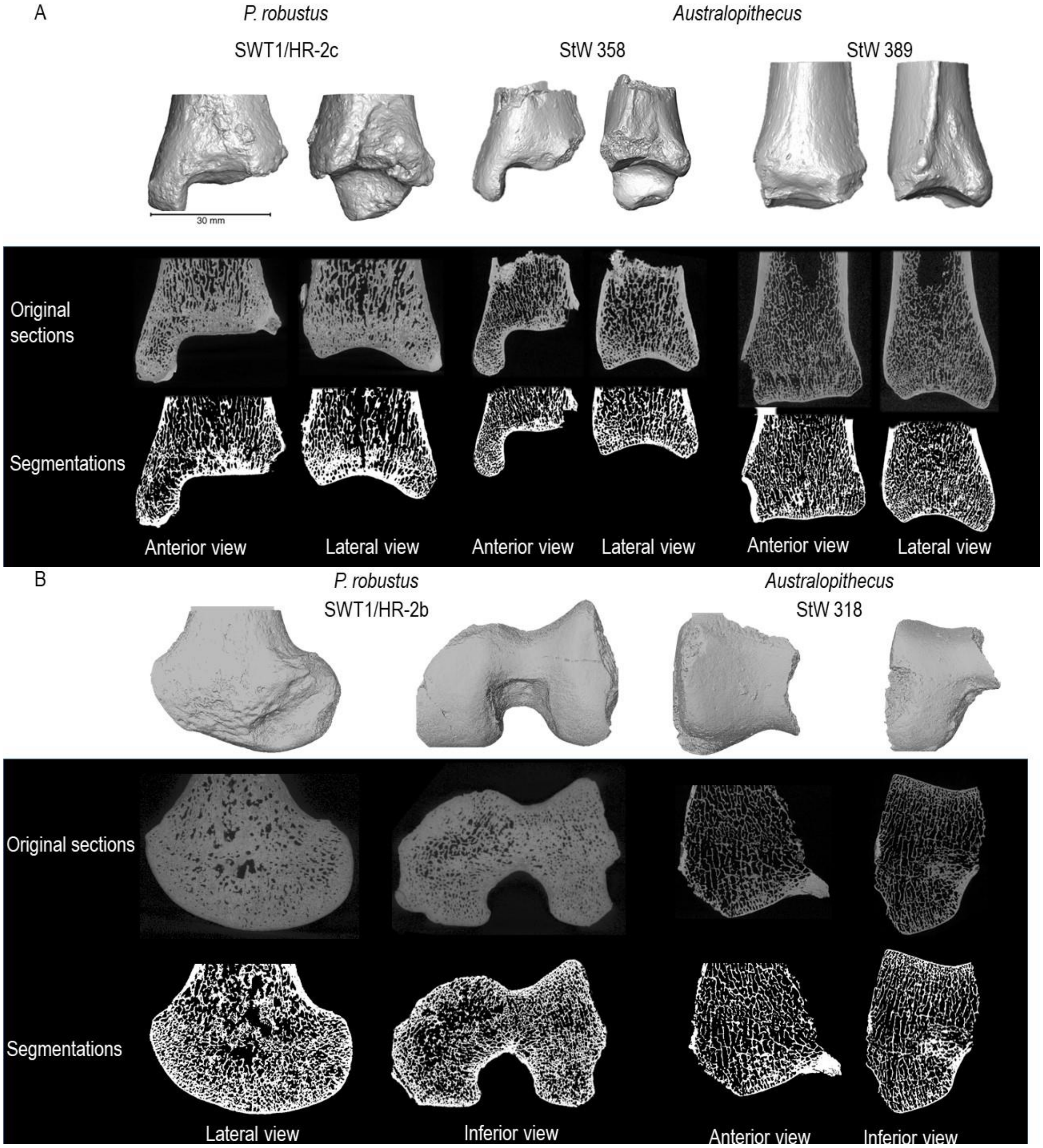

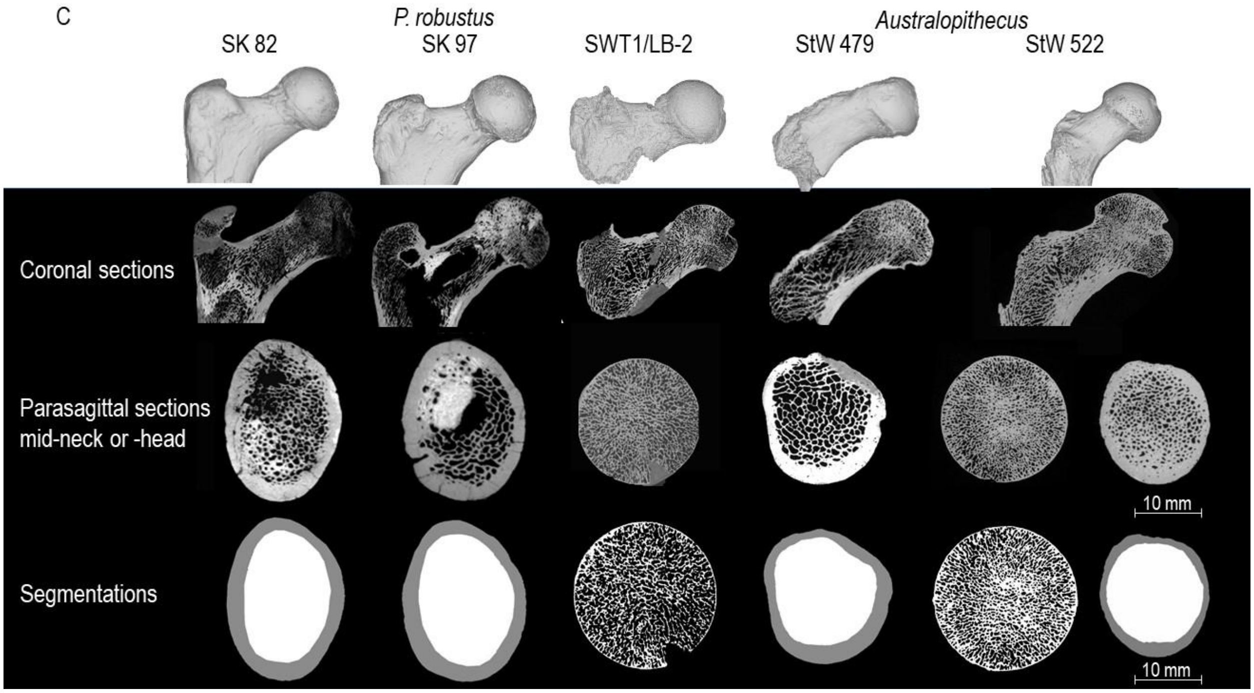
C
P. robustus
Australopithecus
SK 82
SK 97
SWT1/LB-2
StW 479
StW 522
Coronal sections
Parasagittal sections
mid-neck or -head
Segmentations
10 mm
10 mm

Fig. S3. Results of principal component analysis of the distribution of the relative bone volume fraction of the distal tibia in *Australopithecus*, *P. robustus*, modern humans, *Pan* and *Gorilla*. The extreme configurations of the first two components are included, showing the main regions of the bone that drive the variability observed in these axes. Density plots show the distribution of within-group Euclidean distances calculated from the first three principal components for modern humans, *Pan*, and *Gorilla*. Each dashed vertical line represents one pairwise Euclidean distance between a *P*. *robustus* specimen and an *Australopithecus* specimen. The position of these dashed lines relative to the extant intraspecific distributions illustrates whether fossil interspecific distances fall within or exceed the range of extant within-group variation.

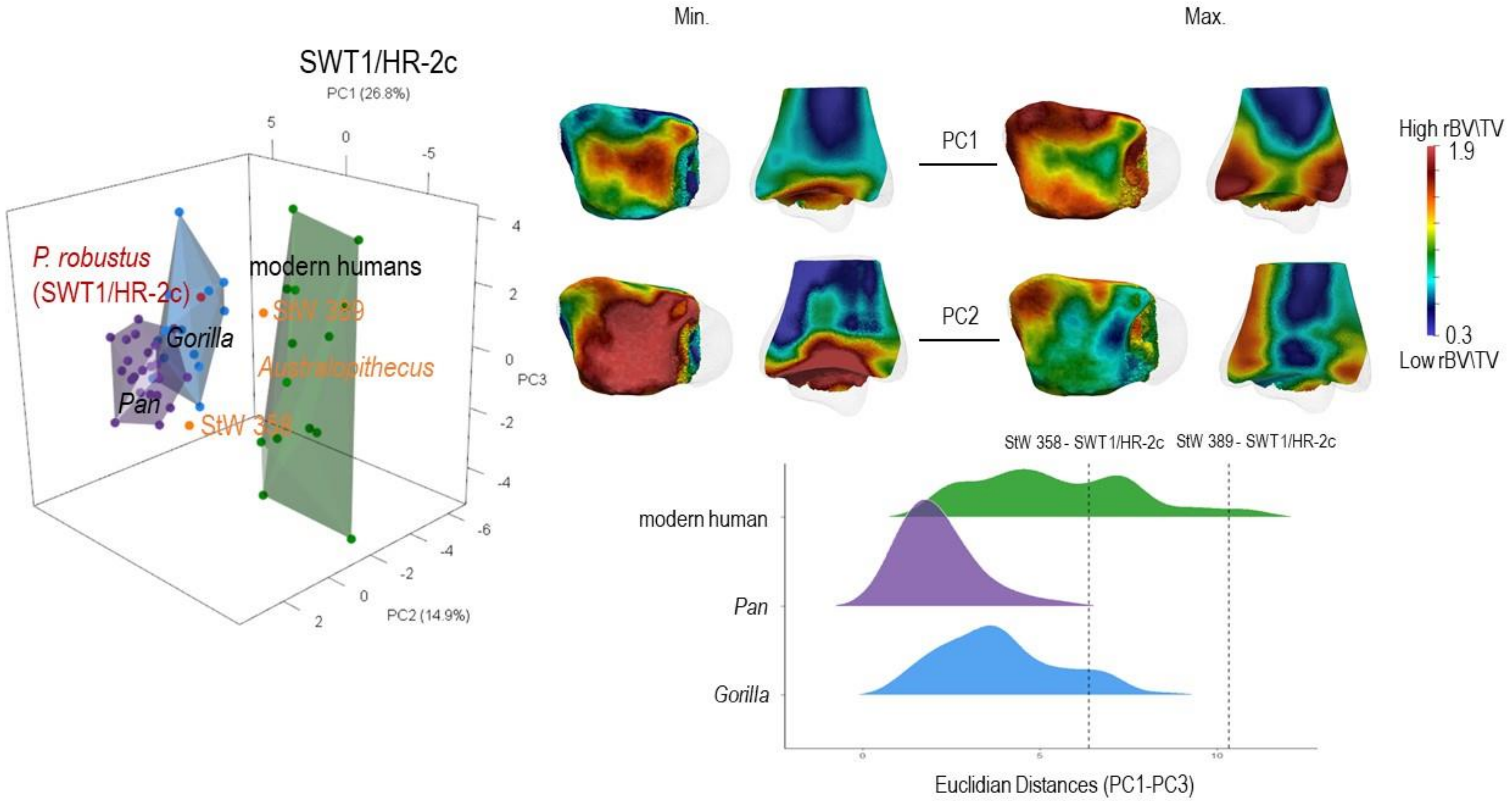

Fig. S4. Bi-variate plot of the first two components of the canonical variate analysis conducted on the first five principal components of the principal component analyses of trabecular bone distribution of the distal tibia in modern humans, *Pan*, *Gorilla* with the projected *Australopithecus* and *P. robustus* individuals. In the table, the cross-validated typicality probabilities of the fossil to be assigned to each of the extant taxa based on canonical variates are presented.

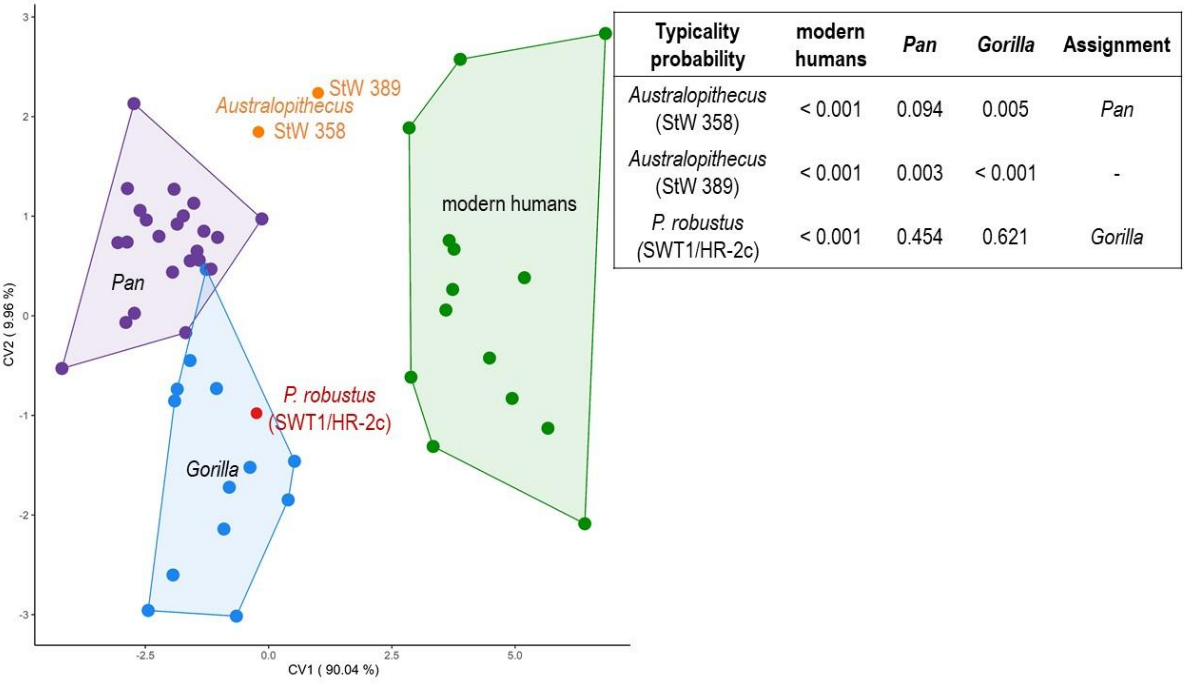

Typicality probability	modern humans	*Pan*	*Gorilla*	Assignment
Australopithecus (StW 358)	< 0.001	0.094	0.005	*Pan*
Australopithecus (StW 389)	< 0.001	0.003	< 0.001	-
P. robustus (SWT1/HR-2c)	< 0.001	0.454	0.621	*Gorilla*

Fig. S5. 3D plot of the first three components of the principal component analysis of the distribution of the relative bone volume fraction of the distal femur in *Australopithecus*, *P. robustus*, modern humans, *Pan* and *Gorilla*. The extreme configurations of the first two components are included, showing the main regions of the bone that drive the variability observed in these axes. Density plots show the distribution of within-group Euclidean distances calculated from the first three principal components for modern humans, *Pan*, and *Gorilla*. Each dashed vertical line represents one pairwise Euclidean distance between a *P*. *robustus* specimen and an *Australopithecus* specimen. The position of these dashed lines relative to the extant intraspecific distributions illustrates whether fossil interspecific distances fall within or exceed the range of extant within-group variation.

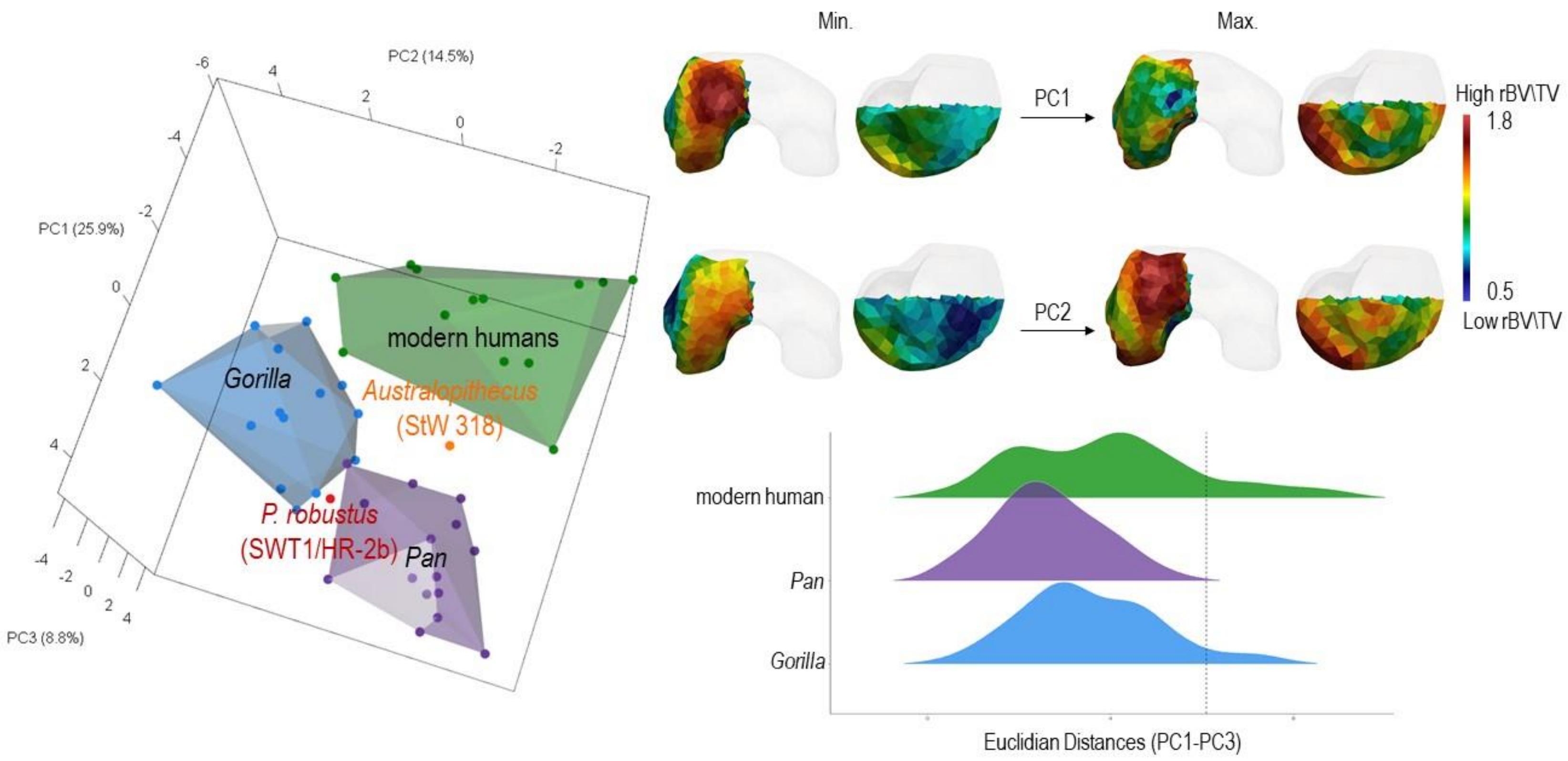

Fig. S6. Bi-variate plot of the first two components of the canonical variate analysis conducted on the first two principal components of the principal component analyses of trabecular bone distribution of the distal femur in modern humans, *Pan*, *Gorilla* with the projected *Australopithecus* and *P. robustus* specimens. The table presents the cross-validated typicality probabilities of each fossil to be assigned to each of the extant taxa based on canonical variates.

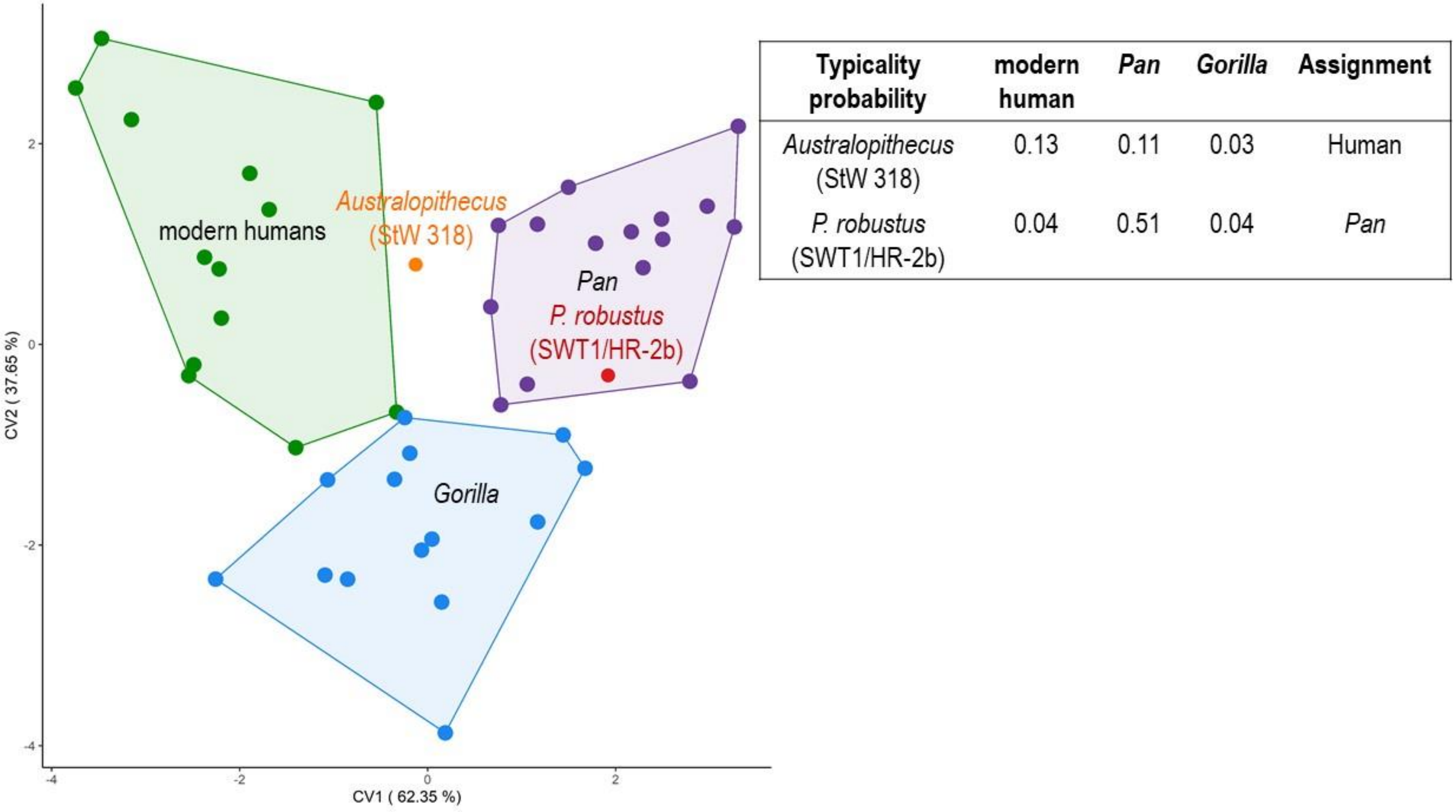

Typicality probability	modern human	*Pan*	*Gorilla*	Assignment
Australopithecus (StW 318)	0.13	0.11	0.03	Human
P. robustus (SWT1/HR-2b)	0.04	0.51	0.04	*Pan*

Fig. S7. Distribution of the relative bone volume fraction (rBV/TV) of the investigated superior region of the femoral head in modern humans, *Pan* and *Gorilla* individuals all presented to same colour scale.

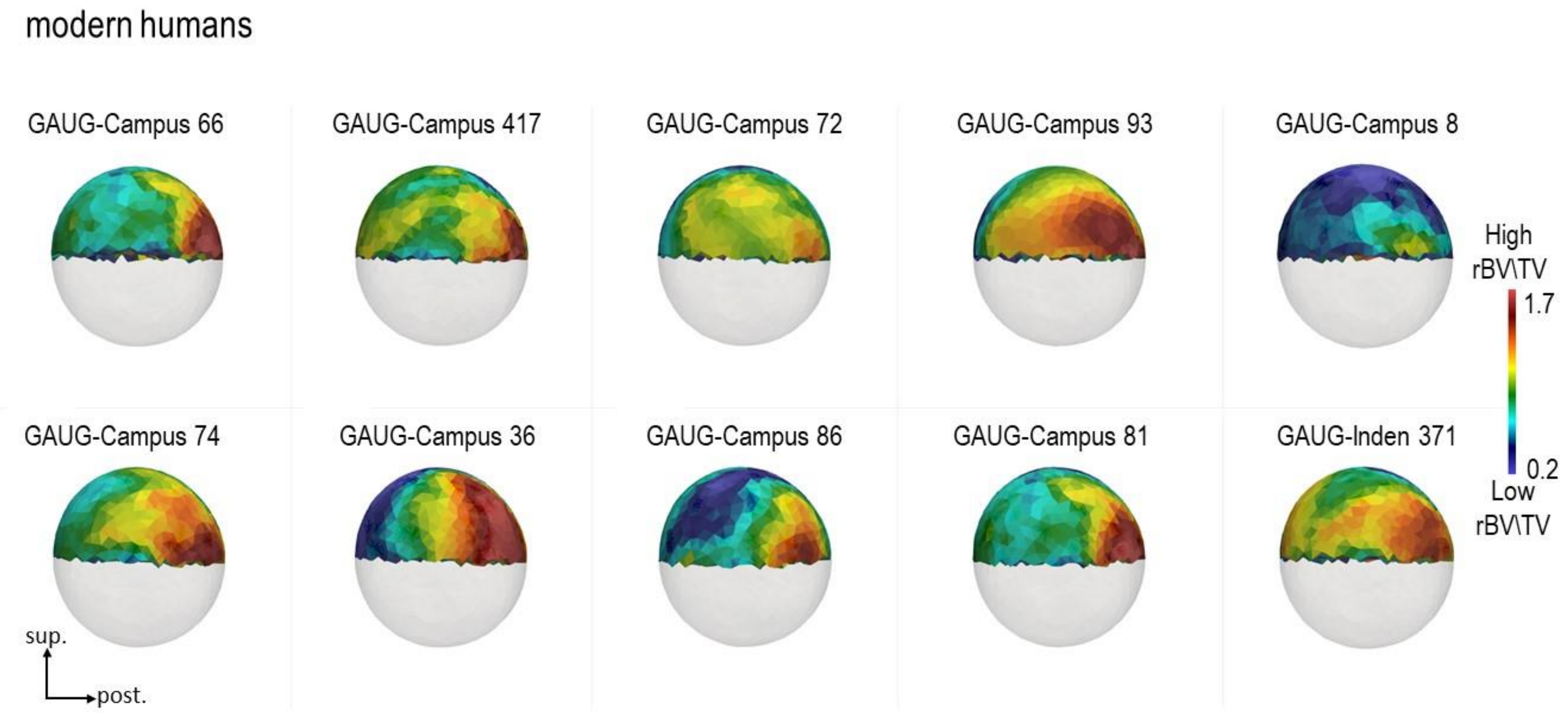

Pan

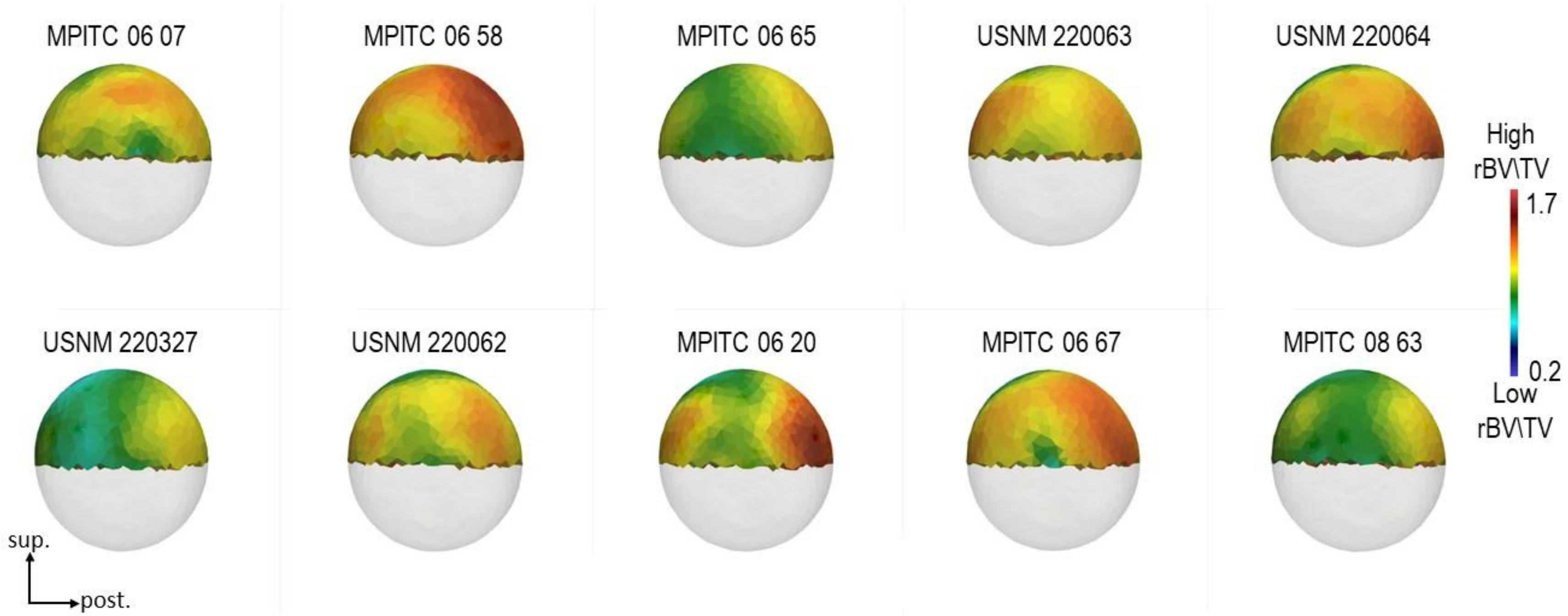

MPITC 06 07
MPITC 06 58
MPITC 06 65
USNM 220063
USNM 220064
USNM 220327
USNM 220062
MPITC 06 20
MPITC 06 67
MPITC 08 63
High rBV\TV
1.7
0.2
Low rBV\TV
sup.
post.

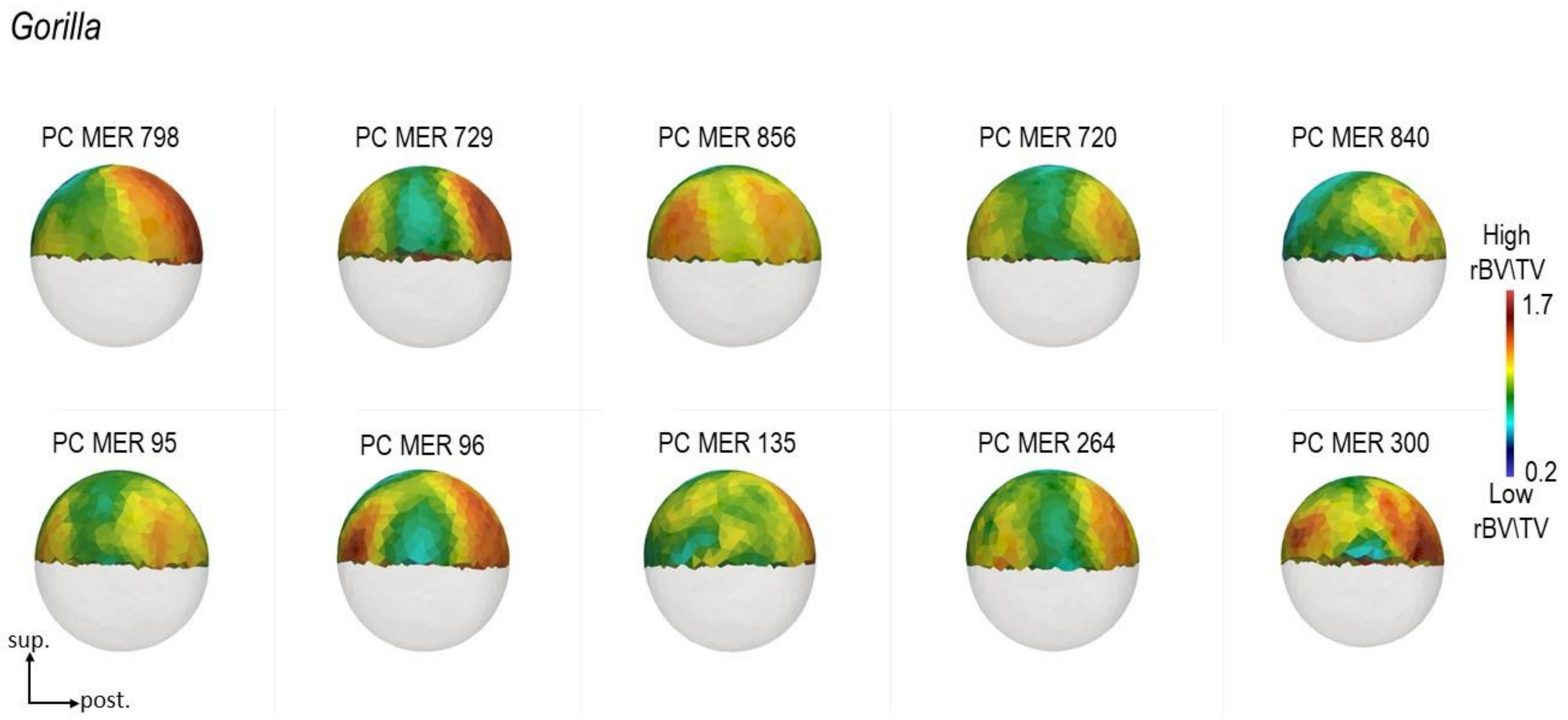
Gorilla
PC MER 798
PC MER 729
PC MER 856
PC MER 720
PC MER 840
PC MER 95
PC MER 96
PC MER 135
PC MER 264
PC MER 300
High
rBV\TV
1.7
0.2
Low
rBV\TV
sup.
post.

Fig. S8. 3D plot of the first three components of the principal component analysis of the distribution of the relative bone volume fraction (rBV/TV) of the superior portion of the femoral head in *Australopithecus*, *P. robustus*, modern humans, *Pan* and *Gorilla*. The extreme configurations of the first two components are included, showing the main regions of the bone that drive the variability observed in these axes. Density plots show the distribution of within-group Euclidean distances calculated from the first three principal components for modern humans, *Pan*, and *Gorilla*. Each dashed vertical line represents one pairwise Euclidean distance between a *P*. *robustus* specimen and an *Australopithecus* specimen. The position of these dashed lines relative to the extant intraspecific distributions illustrates whether fossil interspecific distances fall within or exceed the range of extant within-group variation.

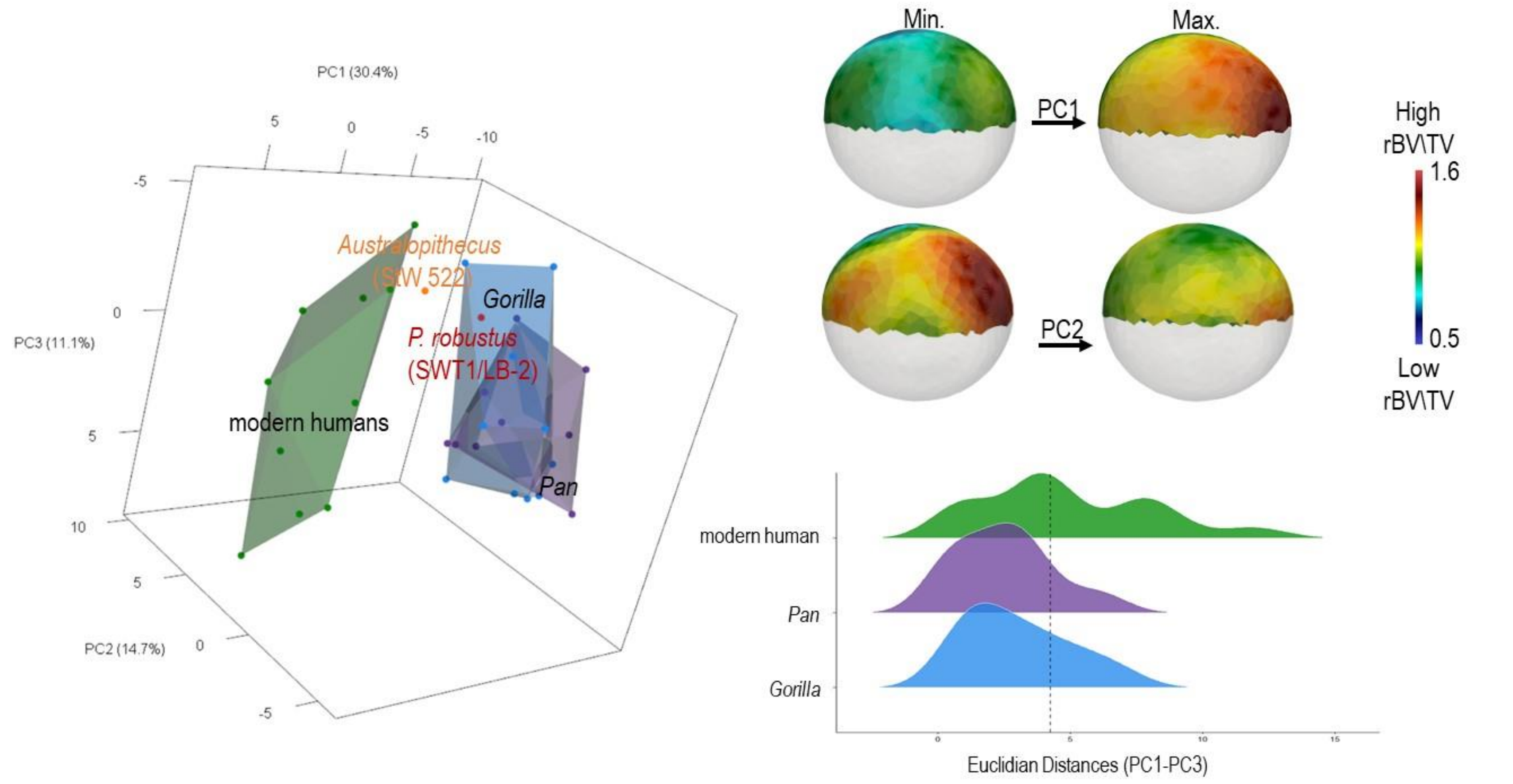

Fig. S9. Bi-variate plot of the first two components of the canonical variate analysis conducted on the first fifth principal components of the principal component analyses of trabecular bone distribution of the femoral head in modern humans, *Pan*, *Gorilla* with the projected *Australopithecus* and *P.* robustus specimens. The table presents the cross-validated typicality probabilities of each fossil to be assigned to each of the extant taxon based on canonical variates.

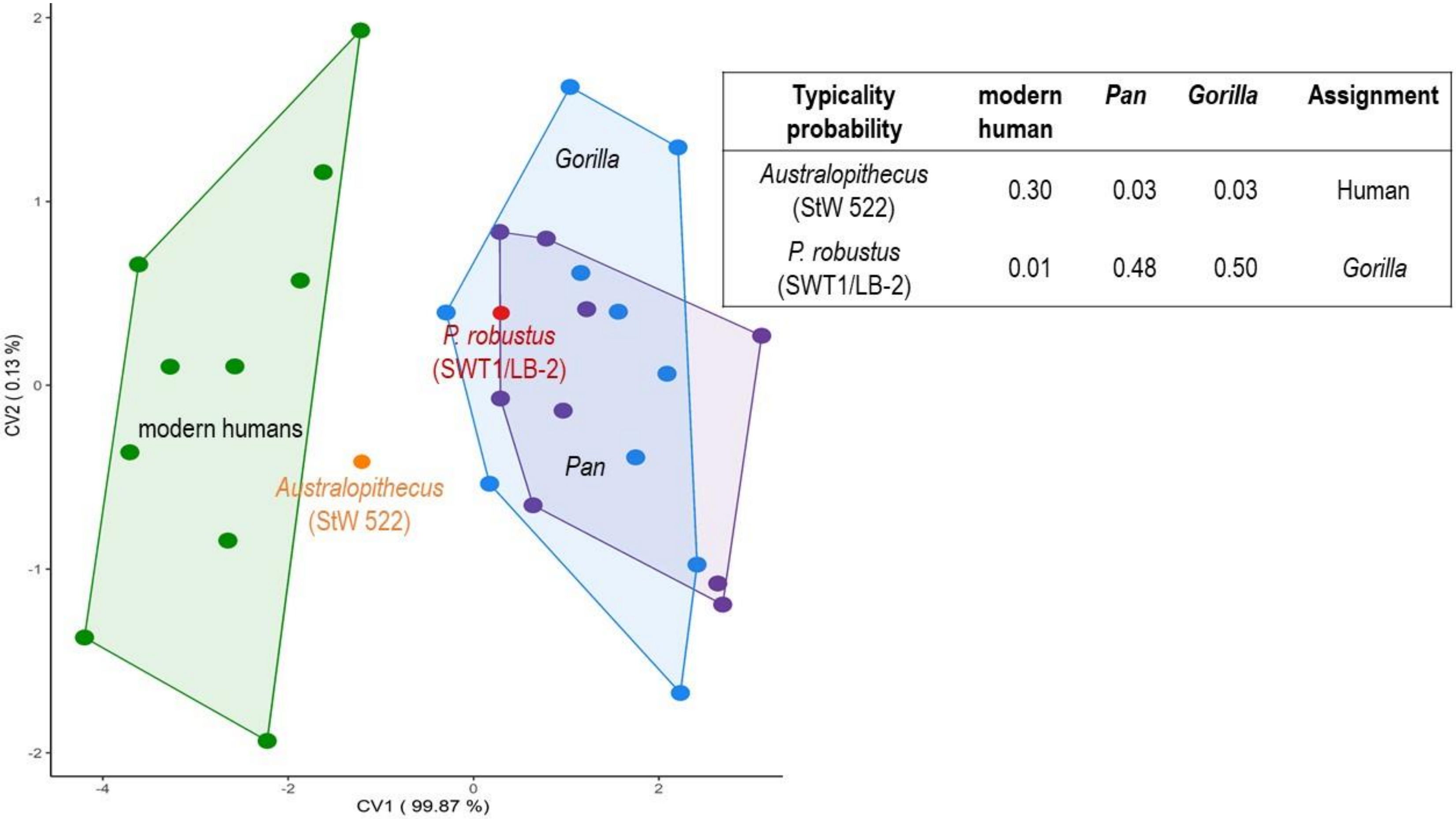

Typicality probability	modern human	*Pan*	*Gorilla*	Assignment
Australopithecus (StW 522)	0.30	0.03	0.03	Human
P. robustus (SWT1/LB-2)	0.01	0.48	0.50	*Gorilla*

Fig. S10. Distribution of the relative cortical thickness (rmsCBT) of the femoral neck of the extant and fossil samples rendered in a chromatic scale from blue (low rmsCBT) to red (high rmsCBT). 'ant.', anterior; 'inf.', inferior; 'post', posterior; 'sup', superior.

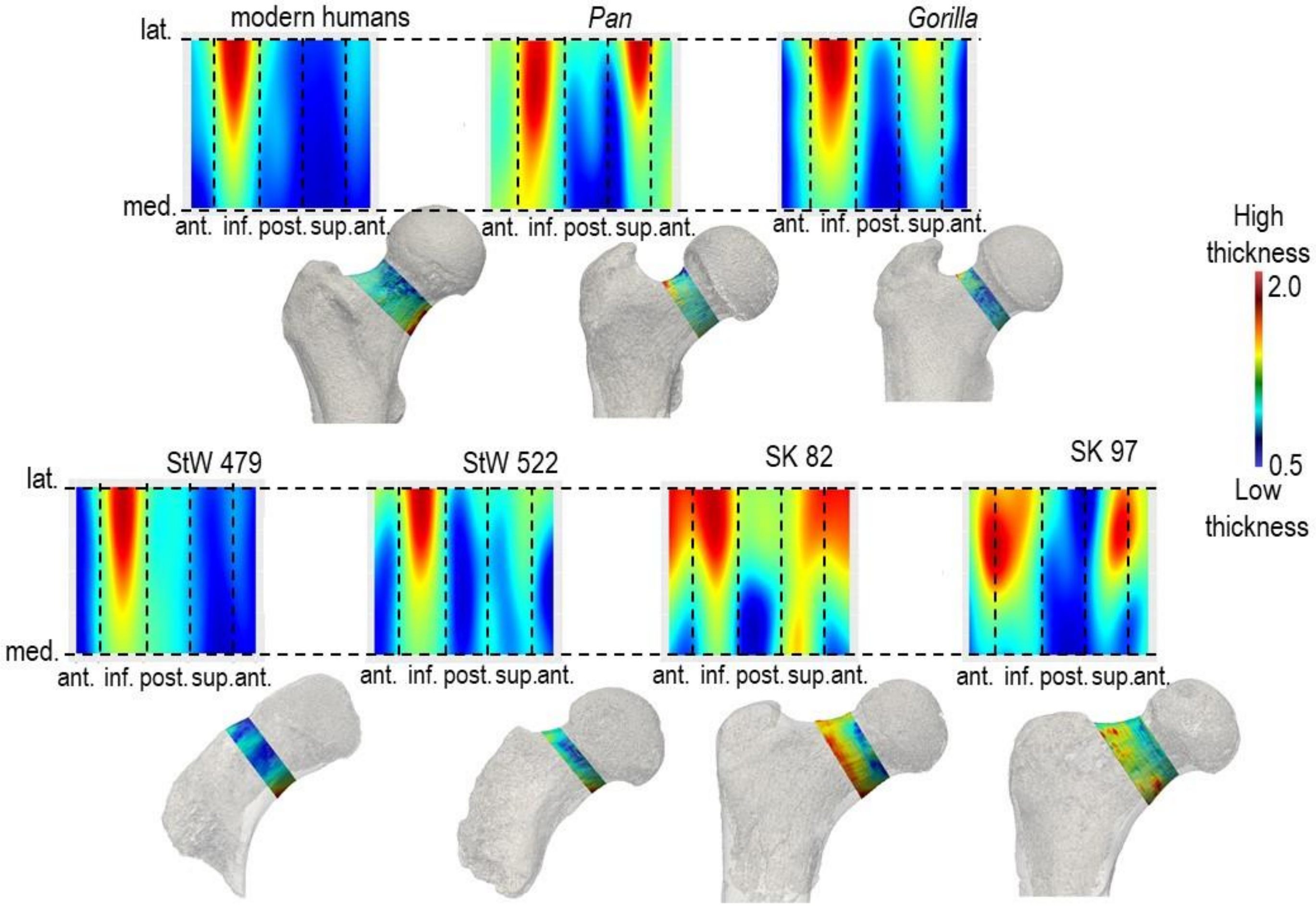

Fig. S11. 3D plot of the first three components of the principal component analysis of the distribution of the relative cortical thickness distribution of the femoral neck in *Australopithecus*, *P. robustus*, modern humans, *Pan* and *Gorilla*. The extreme configurations of the first two components are included, showing the main regions of the bone that drive the variability observed in these axes. Abbreviations the same as in Fig. S11. Density plots show the distribution of within-group Euclidean distances calculated from the first three principal components for modern humans, *Pan*, and *Gorilla*. Each dashed vertical line represents one pairwise Euclidean distance between a *P*. *robustus* specimen and an *Australopithecus* specimen. The position of these dashed lines relative to the extant intraspecific distributions illustrates whether fossil interspecific distances fall within or exceed the range of extant within-group variation.

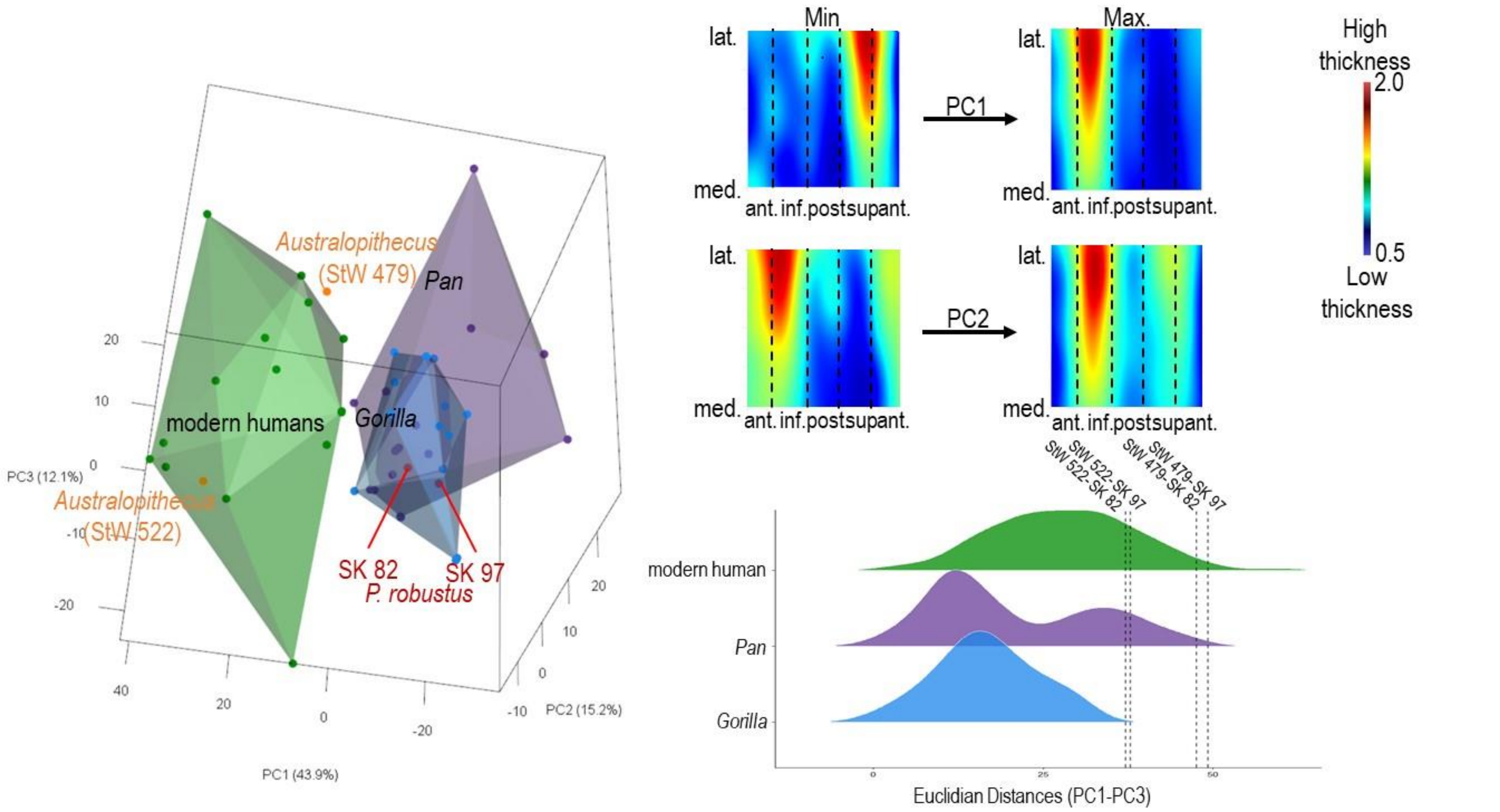

Fig. S12. Bi-variate plot of the first two components of the canonical variate analysis conducted on the first fifth principal components of the principal component analyses of trabecular bone distribution of the femoral head in modern humans, *Pan*, *Gorilla* with the projected *Australopithecus* and *P. robustus* individuals. The table presents the cross-validated typicality probabilities of each fossil to be assigned to each extant taxon based on canonical variates.

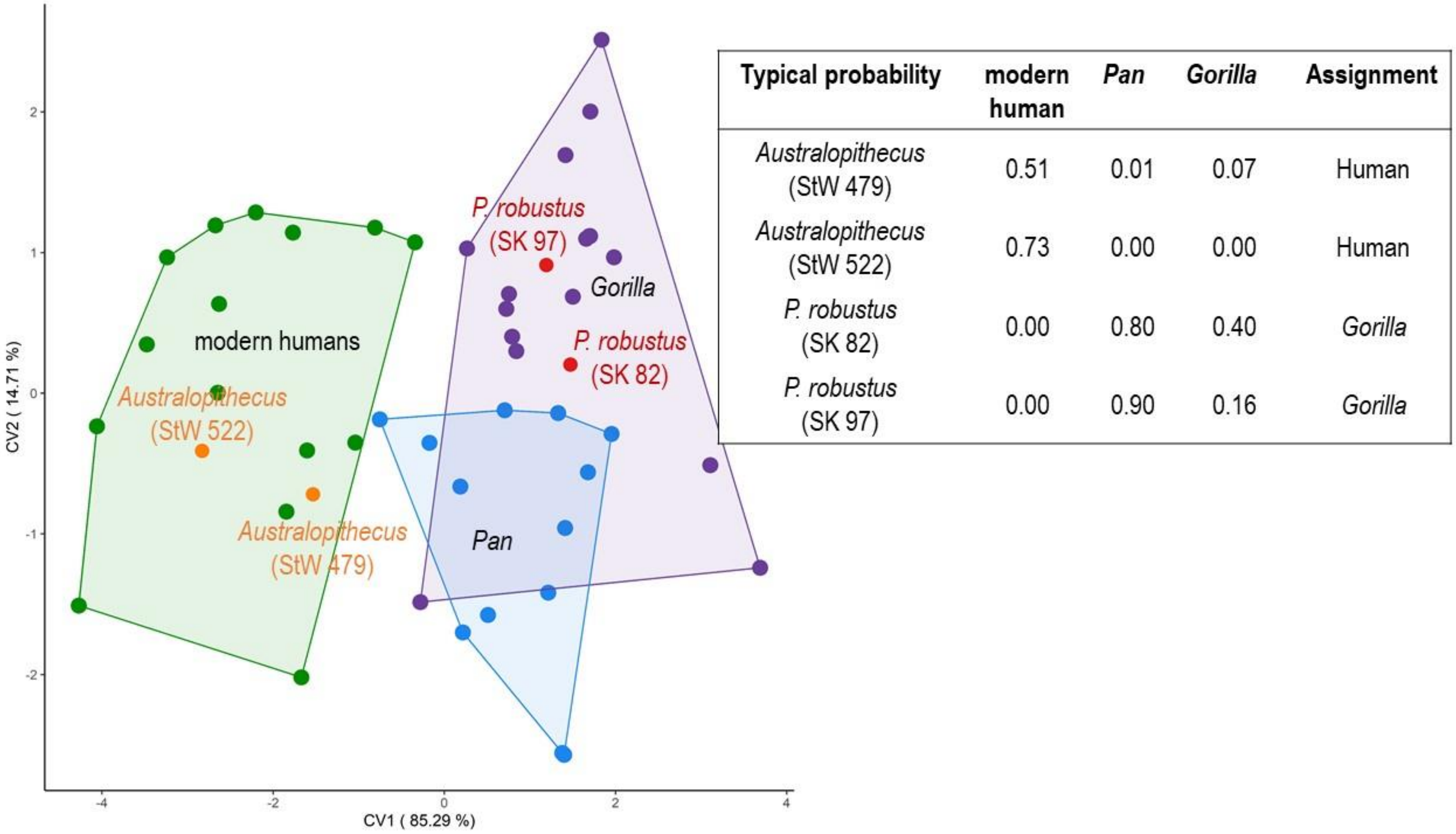

Typical probability	modern human	*Pan*	*Gorilla*	Assignment
Australopithecus (StW 479)	0.51	0.01	0.07	Human
Australopithecus (StW 522)	0.73	0.00	0.00	Human
P. robustus (SK 82)	0.00	0.80	0.40	*Gorilla*
P. robustus (SK 97)	0.00	0.90	0.16	*Gorilla*

Fig. S13. Summary of our results of cortical bone thickness distribution of the femoral neck and trabecular organisation of the femoral head, distal femur and distal tibia of Paranthropus from Swartkrans Member 1 (left row) and Australopithecus from Sterkfontein Member 4 (right row), southern Africa. Individual maps of preserved trabecular bone are shown projected onto the preserved external morphology of each investigated fossil specimen. The results show a combination of adaptations to flexed and abducted hip (thick cortical bone of the superior surface of the femoral neck and anterior trabecular reinforcement of the femoral head), as well as flexed knee (higher trabecular bone density in the posterior surface of the lateral condyle) and ankle joints (trabecular reinforcement in the anterior and posterior margin of the articular surface of the distal tibia). Together these results indicate that climbing, was a more frequent component of the *P. robustus* locomotor repertoire than that of southern African *Australopithecus*

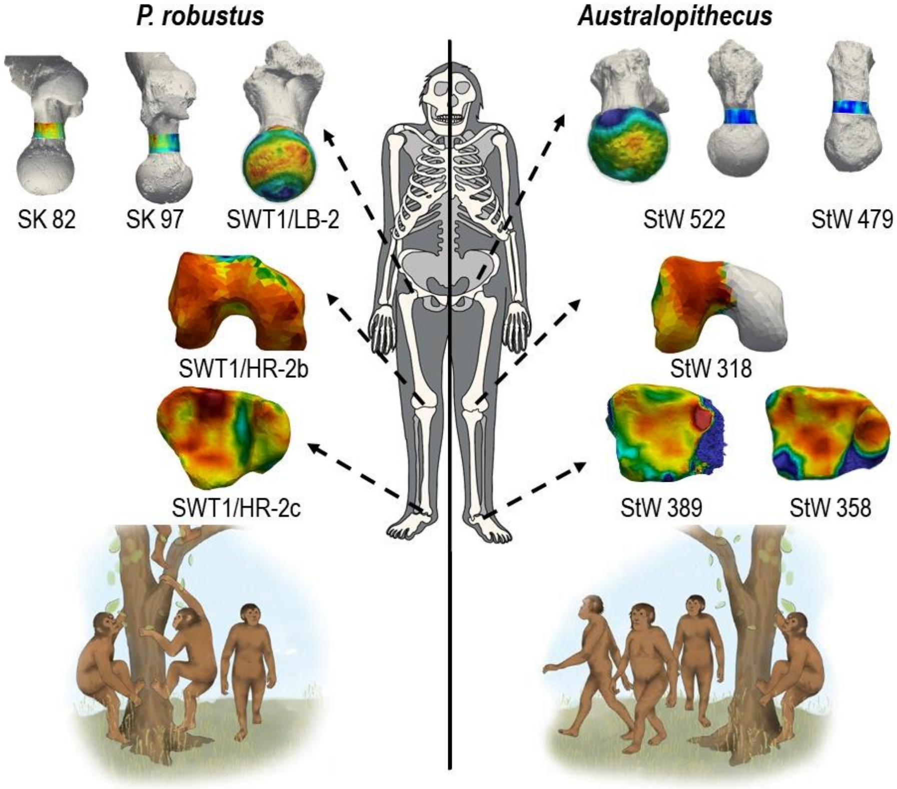

.

Fig. S14. Effect of segmentation method on PCA position and trabecular distribution in the *Australopithecus* distal femur (StW 318). Principal component analysis (PC1 vs. PC2) based on rBV/TV distribution, showing extant taxa (modern humans, *Pan*, *Gorilla*) and fossil specimens. The positions of StW 318 segmented using Mia Clustering (StW 318 MIA) and Dragonfly (StW 318 Dragonfly) are indicated. Euclidean distances (PC1–PC3) among all specimens, highlighting the distance between the two segmentations of StW 318 (red). The segmentation pair ranks 4th out of 990 pairwise comparisons (0.4th percentile). Three-dimensional renderings of the distal femur showing rBV/TV distribution for StW 318 segmented using Dragonfly (left) and Mia Clustering (right). Color scale indicates low (blue) to high (red) relative bone volume fraction.

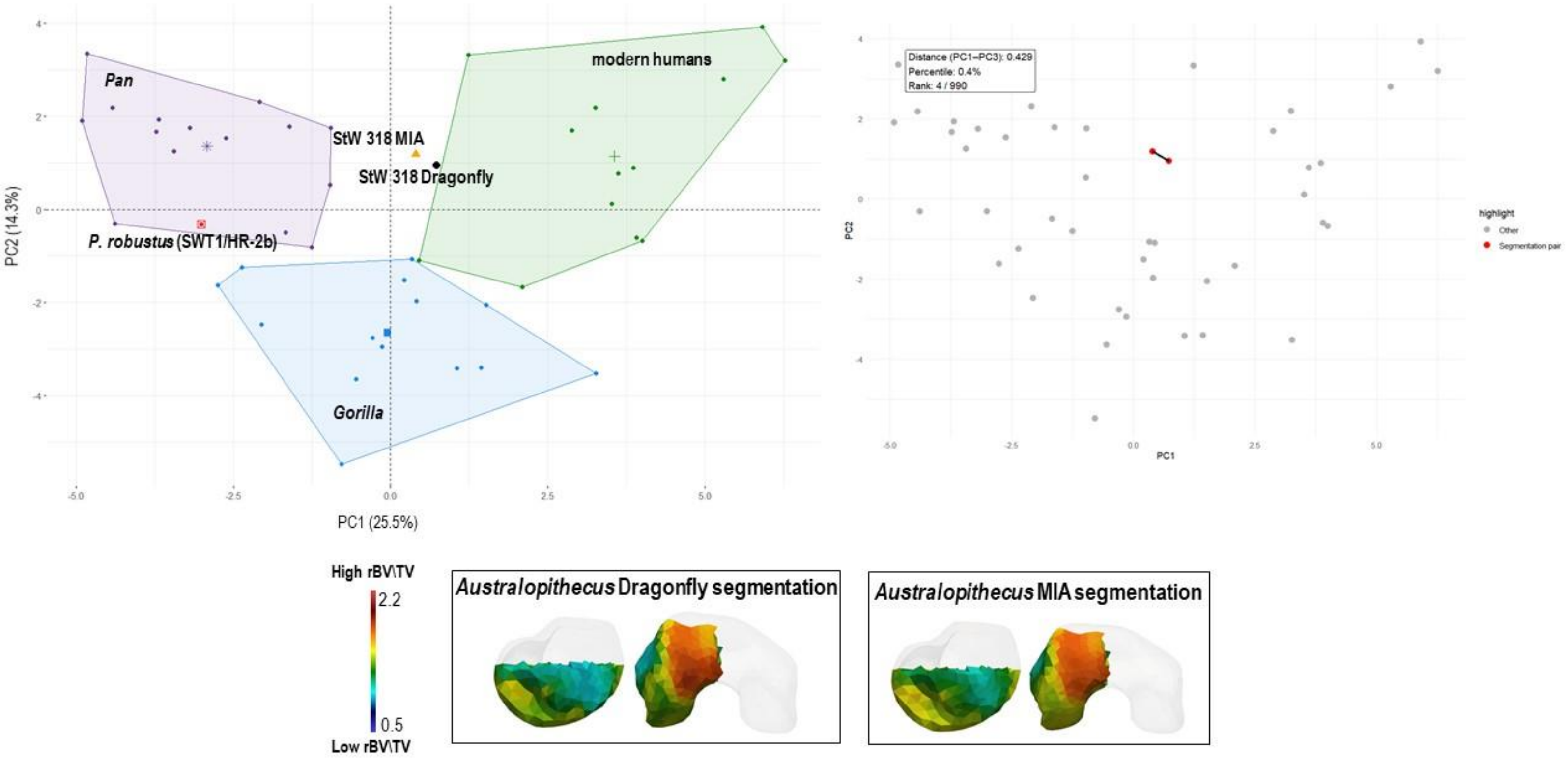

Tables

Table S1. Details of the fossil sample used in this study. Excavation square and depth below datum are provided for Sterkfontein M4 specimens.

**Specimen**	**Side**	**Taxon**	**Chronology/Provenance**	**Collection[a]**	**Scan location[b]**	**Voxel size (μm)**	**Investigated region**
SK 82	R	*Paranthropus robustus*	Early Pleistocene/Swartkrans Member 1, South Africa	Ditsong	NECSA	79	Femoral neck
SK 97	R	*Paranthropus robustus*	Early Pleistocene/Swartkrans Member 1, South Africa	Ditsong	NECSA	70	Femoral neck
StW 318	R	Sterkfontein *Australopithecus*	Plio-Pleistocene/Sterkfontein Member 4, South Africa (U/49; 24’0’’ – 25’0’’)	ESI	ESI		Distal femur
StW 358	L	Sterkfontein *Australopithecus*	Plio-Pleistocene/Sterkfontein Member 4, South Africa (P/43; 13’6’’ – 14’6’’)	ESI	ESI	32	Distal tibia
StW 389	L	Sterkfontein *Australopithecus*	Plio-Pleistocene/Sterkfontein Member 4, South Africa (O/47; 18’10’’ – 19’9’’)	ESI	ESI	32	Distal tibia
StW 479	R	Sterkfontein *Australopithecus*	Plio-Pleistocene/Sterkfontein Member 4, South Africa (V/43; 23’2’’ – 24’2’’)	ESI	ESI	30	Femoral neck
StW 522	L	Sterkfontein *Australopithecus*	Plio-Pleistocene/Sterkfontein Member 4, South Africa (P/45; 26’1’’ – 27’1’’)	ESI	MPIEVA	33	Femoral neck/head
SWT1/HR-2b	L	*Paranthropus robustus*	Early Pleistocene/Swartkrans Member 1, South Africa	ESI	NECSA	28	Distal femur
SWT1/HR-2c	L	*Paranthropus robustus*	Early Pleistocene/Swartkrans Member 1, South Africa	ESI	NECSA	28	Distal tibia
SWT1/LB-2	R	*Paranthropus robustus*	Early Pleistocene/Swartkrans Member 1, South Africa	ESI	NECSA	46	Femoral head

[a]ESI = Evolutionary Studies Institute of the University of the Witwatersrand, Johannesburg, South Africa;
[b]NECSA = MIXRAD microfocus X-ray tomography facility (Nikon XTH 225 ST Metris equipment) at the South African Nuclear Energy Corporation SOC Ltd, Pelindaba, South Africa; ESI = microfocus X-ray tomography facility (Nikon XT H225/320L industrial XCT system) at the Evolutionary Studies Institute of the University of the Witwatersrand, Johannesburg, South Africa; MPIEVA = microfocus X-ray tomography facility (BIR ACTIS 225/300 industrial microCT scanner) at the Department of Human Evolution, Max Planck Institute for Evolutionary Anthropology, Leipzig, Germany

Table S2. Details of the comparative sample used in the present study.

Taxon	Investigared region	n	Sex	Age	Context	Collection[a]	Scan location[b]	Voxel size
modern humans	Femoral head	10	3F, 5M, 2NA	A	likely sedentary	GAUG	MPI-EVA	65-70
	Femoral neck	15	8F, 7M	A	likely sedentary, Hunter-gatherers	GAUG, PBC, McGregor	MPI-EVA, NECSA	29-69
	Distal femur	15	1M, 14NA	A	likely sedentary	NGA, NGB, UT	MPI-EVA, Univ. Kent	30-37
	Distal tibia	14	5F, 9M	A	likely sedentary	GAUG, SMU	MPI-EVA, NECSA	30-36
Pan troglodytes	Femoral head	10	5F, 5M	A	wild	MPI-EVA, USNM	MPI-EVA	40-50
	Femoral neck	16	8F,7M,1NA	A	wild	MPI_EVA, USNM	MPI-EVA	30-35
	Distal femur	15	8F, 7M	A	wild	MPI-EVA	MPI-EVA	29-30
	Distal tibia	23	13F, 10M	A	wild	MPI-EVA, AMNH	MPI-EVA, AMNH	26-30
Gorilla gorilla	Femoral head	10	6F, 4M	A	wild	PCM	CBC	50-70
	Femoral neck	14	7F,7M	A	wild	PCM, AMNH	CBC, AMNH	45-75
	Distal femur	14	7F, 8M	A	wild	PCM	Univ. Kent	48-58
	Distal tibia	13	4F, 3M	A	wild	PCM, AMNH	CBC, AMNH	31-73

[a]GAUG = Georg-August-Universität Göttingen, Germany; PBC = Pretoria Bone Collection, Pretoria, South Africa; McGM = McGregor Museum, Kimberley, South Africa; NGA = St. Gregory's Priory, Skeletal Biology Research Centre, University of Kent, UK; NGB = St. Gregory's Priory, Skeletal Biology Research Centre, University of Kent, UK; UT = W.M. Bass femoral collection, Forensic Anthropology Centre, University of Tennessee, USA; SMU = Human Bone Collection, Sefako Makgatho Health Sciences University, Pretoria, South Africa; MPI-EVA = Max Planck Institute for Evolutionary Anthropology, Leipzig, Germany; USNM = Zoologische Staatssammlung München, Germany; AMNH = Department of Mammalogy, American Museum of Natural History, New York, USA; PCM = Powell-Cotton Museum, Birchington-on-Sea, UK

[b]MPIEVA = microfocus X-ray tomography facility (BIR ACTIS 225/300 industrial microCT scanner) at the Department of Human Evolution, Max Planck Institute for Evolutionary Anthropology, Leipzig, Germany; NECSA = MIXRAD microfocus X-ray tomography facility (Nikon XTH 225 ST Metris equipment) at the South African Nuclear Energy Corporation SOC Ltd, Pelindaba, South Africa; Univ. Kent = Imaging Centre for Life Sciences (Diondo d1 Micro-CT Scanner) at the University of Kent, Canterbury, UK; AMNH = Microscopy and Imaging Facility (2010 GE phoenix v|tome|x s240 system) at the American Museum of Natural History, New York, USA; CBC = Cambridge Biotomography Centre (Nikon XT 225 ST Metris scanner) in the Department of Zoology at the University of Cambridge, Cambridge, UK

Table S3. *P*-values of the pairwise permutational MANOVA tests with Bonferroni corrections run on the Euclidean distance matrices of the first three principal components scores calculated of the principal component analyses of the relative bone volume fraction distribution of the distal tibia, distal femur, femoral head and femoral neck of modern humans, *Pan* and *Gorilla*. Significant differences ($p < 0.05$) are in bold.

**Distal tibia**	*Pan*	*Gorilla*
modern human	**0.010**	**0.010**
Pan	-	**0.010**
**Distal femur**	*Pan*	*Gorilla*
modern human	**0.003**	**0.003**
Pan	-	**0.003**
**Femoral head**	*Pan*	*Gorilla*
modern human	**< 0.001**	**< 0.001**
Pan	-	**< 0.001**
**Femoral neck**	*Pan*	*Gorilla*
modern human	**< 0.001**	**< 0.001**
Pan	-	**< 0.001**

SI Appendix, II

What we know: Response to loading in bone and bone volume fraction (BV/TV)

Since the 19th century, the correlation between mechanical forces and bone mass has been well established, commonly referred to as 'Wolff's law' or more recently 'bone functional adaptation' (e.g., 86-88). This principle states that cortical and trabecular bone tissues respond to mechanical stimuli by adjusting (i.e., modeling) their architecture to improve their mechanical function to withstand habitual *in vivo* loading conditions (89-91, and references therein). Wolff's law refers to a very strict relationship between bone structure and mechanical loading, whereas bone functional adaptation is a more open/flexible concept, acknowledging the other factors that influence bone structure (92,93). The cellular biological process by which this adjustment to loading occurs is mechanotransduction. Mechanical stimuli are thought to be translated via mechanosensitive signaling pathways into biochemical responses carried out by bone cells. Osteoblasts (responsible for bone deposition), osteoclasts (responsible for bone resorption), and osteocytes (mediating their action) receive and respond to these diverse mechanical stimuli (94-96, and references therein).

Bone volume fraction (BV/TV, sometimes also referred to as 'bone density') reflects the proportion of trabecular bone volume relative to total volume in a given space. Greater bone volume fraction (BV/TV) has been shown to positively correspond with higher levels of loading (97,98). In this context, levels of loading refer to magnitudes of stress (i.e., roughly speaking, the mechanical forces applied to the bone per unit area) and the frequency (i.e., how often and how long these forces are applied over time) (99). Thus BV/TV differs from, and should not to be confused with, BMD (bone mineral density). Typically, we measure BV/TV only in high-resolution images (microCT scans or histomorphometry), where the bone tissue can be viewed as a homogeneous material and is assumed to be mineralized to the same degree everywhere. It directly describes the microstructural organization of bone and is highly sensitive to the architecture of trabecular bone, including trabecular thickness, spacing/separation, and connectivity. In contrast, BMD refers to the mass of mineral per unit of bone volume. It reflects how much mineralized tissue is present and how densely it is mineralized. BMD is typically measured using dual-energy X-ray absorptiometry (DXA) or quantitative CT. However, it provides only a global measure of mineral content and does not account for how bone tissue is distributed internally. Two bones with identical BMD values may have very different internal architectures and, therefore, different mechanical properties (see differences between BV/TV and BMD in 100).

In addition, in trabecular bone, individual struts have been experimentally shown to preferentially align along experienced load vectors (97,101). This alignment can be measured using, for instance, fabric tensors (102), which is a mathematic tool to quantify how trabeculae are directionally organized. Building on this, the degree of preferential alignment or, a.k.a, degree of anisotropy (DA) is thought to measure how well bone has adapted to a load vector (i.e., the direction and magnitude of habitual load). BV/TV and fabric tensors are some of the most biomechanically informative aspects of trabecular architecture (103-106). For example, 106 found that approximately 88% of trabecular stiffness can be explained by BV/TV, with fabric tensors accounting for an additional ~10%.

A large literature of experimental work in animal models, case studies, and sports and clinical research further confirms the plasticity of trabecular bone in response to loading, notably highlighting the short-term plastic responses in BV/TV (97, 107, 108). For example, experimental analyses across different locomotor regimes have been conducted in birds (e.g., 109,110), rodents (e.g., 111,112), bovids (113) and sheep (97), examining skeletal joints such as the hip, knee, and ankle (see review in 6). These studies demonstrate that trabecular bone responds rapidly to altered mechanical environments, with localized increases in BV/TV following increased loading stimulus (e.g., 97; 107) and significant decreases under conditions of reduced loading or disuse (e.g., 114). Sports-related studies similarly show that trabecular architecture adapts to habitual loading regimes: athletes exposed to repetitive high-impact loading display site-specific increases in BV/TV within a bone (115), whereas immobilization, injury, or degenerative conditions result in measurable losses of BV/TV (107). Similarly, bedridden patients exhibit significant decreases in trabecular bone density, supporting the strong dependence of high BV/TV on mechanical stimulation (116).

Why is the spatial distribution of BV/TV important?

Bone modeling is inherently local (117). Forces acting on part of a joint surface generate localized increases in BV/TV, producing heterogeneous distributions throughout the whole joint and the bone. This local resolution of bone modeling has been tested with *in vivo* micro-computed tomography in mice showing bone formation (resulting in higher BV/TV) and resorption (resulting in lower BV/TV) at the tissue level in response to high local mechanical stress and low local stress based on finite element models, respectively (107,118).
Some of the first quantitative approaches to analyzing the distribution of BV/TV within a bone or joint of interest used manually-positioned volumes of interest (VOIs) to sample a subsection(s) of trabecular structure. These VOI methods were useful at a time when computational power for coping with large highresolution data sets was much more limited (see review in 119). However, VOI-based methods risk obscuring localized functional adaptations, may miss functional signals by not analyzing the entire trabecular network, suffer from homology issues across morphologically diverse taxa, and lead to averaging of functionally significant heterogeneity by generating single values (see reviews in 119,120). In recent years, more holistic approaches have been developed to overcome these limitations (121; 122). Most recently, the implementation of cHMA (see methods section in 123), using a deformation-based registration approach to measure the entire trabecular network in each bone with sets of overlapping VOIs, enables a homologous statistical comparison of BV/TV (and relative BV/TV, see below) distribution patterns. Therefore, rather than simply quantifying the overall amount of trabecular bone present in a skeletal element or epiphysis quantifying the *spatial distribution* of BV/TV using holistic approaches allows for the identification of bone regions that were likely subjected to relatively higher mechanical loading (magnitude and frequency, see above) compared other regions within the bone or epiphysis (124).

The importance of spatial BV/TV distribution for reconstructing joint loading conditions has also been emphasized by (125), who applied inverse bone remodeling (IBR) to predict physiological loading at the human hip joint. The goal of IBR is to identify the loading history that best reproduces a state of bone modeling equilibrium throughout the entire bone. Using a model that includes spatial BV/TV distribution allowed predictions of plausible peak hip joint loads consistent with in vivo measurements of physiological loads in patients with instrumented prostheses, whereas homogeneous models were unable to predict meaningful loading directions.

Including the trabecular alignment (via fabric tensors) only slightly improved the predictions compared to the models using only BV/TV distributions (Fig. S15).

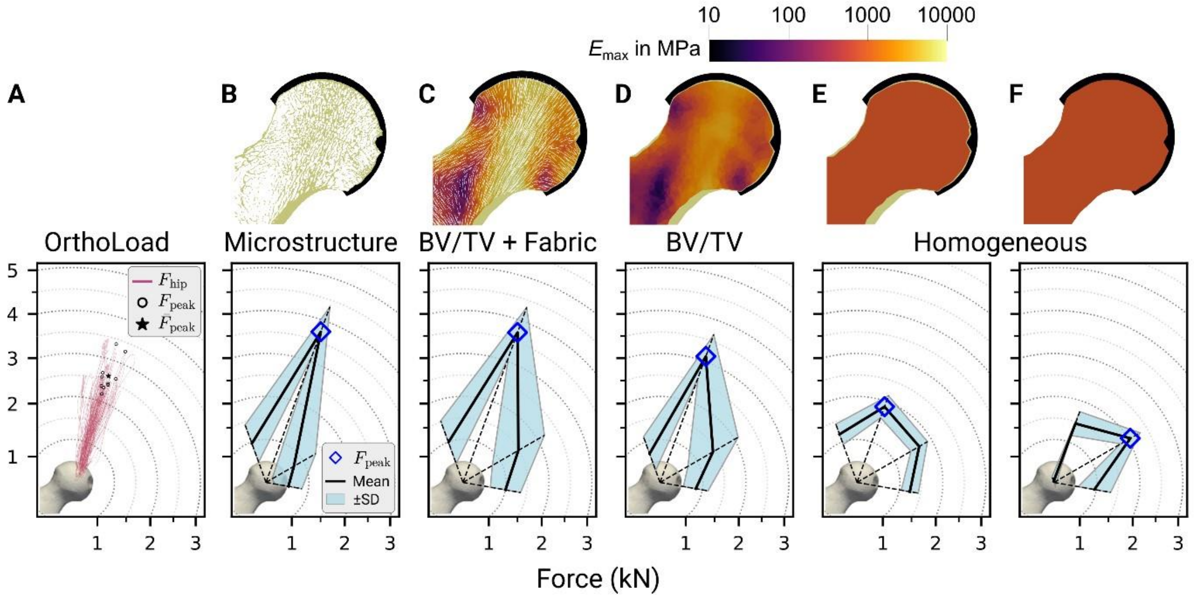

Fig. S15. Inverse bone remodeling of the human hip joint. From left to right: (A) Measured hip joint force vectors from the OrthoLoad database (126) as a reference. (B) Hip joint loading vector predicted with inverse bone remodelling based on micro-FE simulations, inherently integrating all morphometric parameters. (C) Prediction using only BV/TV and fabric, which still reproduces joint loading patterns well. (D) Prediction using BV/TV spatial distribution alone, which still captures the dominant loading vector. (E and F) When BV/TV distribution is removed and a homogeneous material is used ((E) cortical and trabecular averages, (F) single material), information about joint loading is lost (Image from 125).

<u>Why use relative BV/TV (rBV/TV) to control for intra- and interspecific variation in robusticity?</u>

BV/TV values are influenced not only by local biomechanical loading but also by systemic or demographic factors that impact overall trabecular bone robusticity, such as age, hormonal status, and species-wide physiology. For example, chimpanzees, bonobos, and gorillas tend to exhibit higher BV/TV than humans in several bones across the skeleton (127-131), while recent human populations show reduced BV/TV, possibly reflecting more sedentary lifestyles (132, 133). These systemic differences can confound functional interpretation of raw BV/TV values. Even spatial distributions of BV/TV values may be obscured by systemic differences in BV/TV, as BV/TV values contain both a systemic and a local signal (117; 127). If one bone has substantially greater overall BV/TV, any part of its spatial distribution (even the lowest values not associated with higher mechanical stimuli) will be significantly higher than the highest values of another bone with overall lower BV/TV. In this scenario, it is likely that a difference in spatial distribution would not be found in univariate testing of raw BV/TV values. Similar to how variation in size (another systemic factor) can dominate the first principal component in multivariate shape analysis, this systemic component of BV/TV can explain the majority of variation in raw BV/TV values, especially in comparative, interspecific analyses. Fig. S16 is an example of a Principal Component Analysis (PCA) of BV/TV distribution in the 4th metacarpal of hominoid taxa, where approximately 70% of the variation in BV/TV distribution can be explained by overall raw BV/TV, as shown by the boxplots.

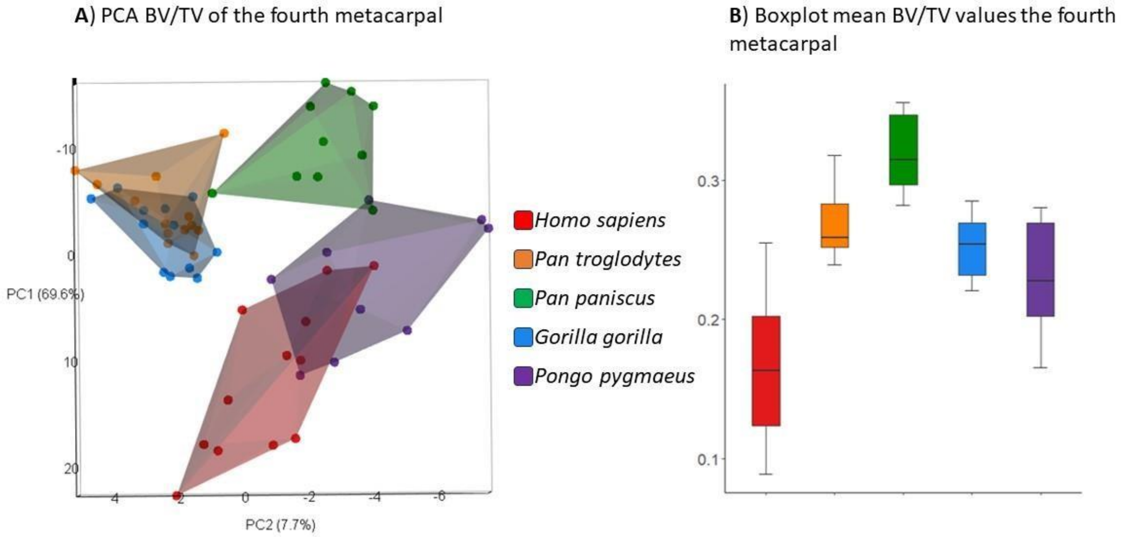

Fig. S16. (A) Principal component analysis (PCA) of the spatial distribution of trabecular bone volume fraction (BV/TV) of the fourth metacarpal (Mc4) across extant hominoid taxa and (B). corresponding boxplot of mean individual BV/TV values. The PCA (left) shows that variation along PC1 on the y-axis (69.6% of total variance) is primarily driven by differences in whole-bone BV/TV, with clear taxon-specific clustering. The boxplots illustrate that taxa with higher PC1 scores in the PCA (notably in *Pan*) also exhibit higher mean BV/TV values (as would be captured by a single average BV/TV value from a large VOI). This correspondence indicates that whole-bone BV/TV is the main driver of the first principal component.

As is standard practice in shape analysis, or any analysis with a confounding scale factor, we focus on relative BV/TV (rBV/TV), standardizing local values by dividing them by the mean BV/TV of each individual. The resulting data show which regions have relatively high or relatively low BV/TV in that particular bone, relative to its mean, and thus largely control for systemic inter- or intraspecific differences in BV/TV values (e.g., 134). This approach therefore focuses on spatial distributional patterns of BV/TV within an individual and allows for meaningful comparison across taxa and individuals. Similar scaling approaches of sBV/TV (135) and z-scores of BV/TV (136-138) have been applied by other groups working on trabecular structure.

<u>What inferences can we make about joint loading in paleoanthropology?</u>

Analysing distributions of rBV/TV is therefore a promising method in paleoanthropology for providing additional information about bone or joint loading and function beyond that which can be inferred from external shape. From the distribution of rBV/TV of a fossil specimen, we can infer— based on the principle of bone functional adaptation— the regions of trabecular bone that were adapted to relatively higher joint loading (higher rBV/TV) compared to regions of lower joint loading (lower rBV/TV) with an epiphysis or bone. In this way, although we cannot infer the exact magnitude or orientation of load, we can identify the location(s) within a joint where loads were likely to have been habitually applied and infer the habitual joint posture (Fig; S17). By comparing patterns of rBV/TV distribution in fossil hominins with those observed in extant primates of known locomotor and tool-use behaviors, we can predict the loaded joint

posture and, ultimately, make inferences about aspects of locomotion and behaviors in fossil hominins.

These inferences are inherently correlative and analogical, with trabecular distributions in fossils being statistically compared with trabecular distribution patterns found in living primates of known behaviours. When inferred joint postures from rBV/TV spatial distributions are consistent with quantified kinematic joint postures during particular behaviours in the living primates (e.g. locomotion, manipulation), we can use this comparative framework to make interpretations about behaviour in extinct individuals.

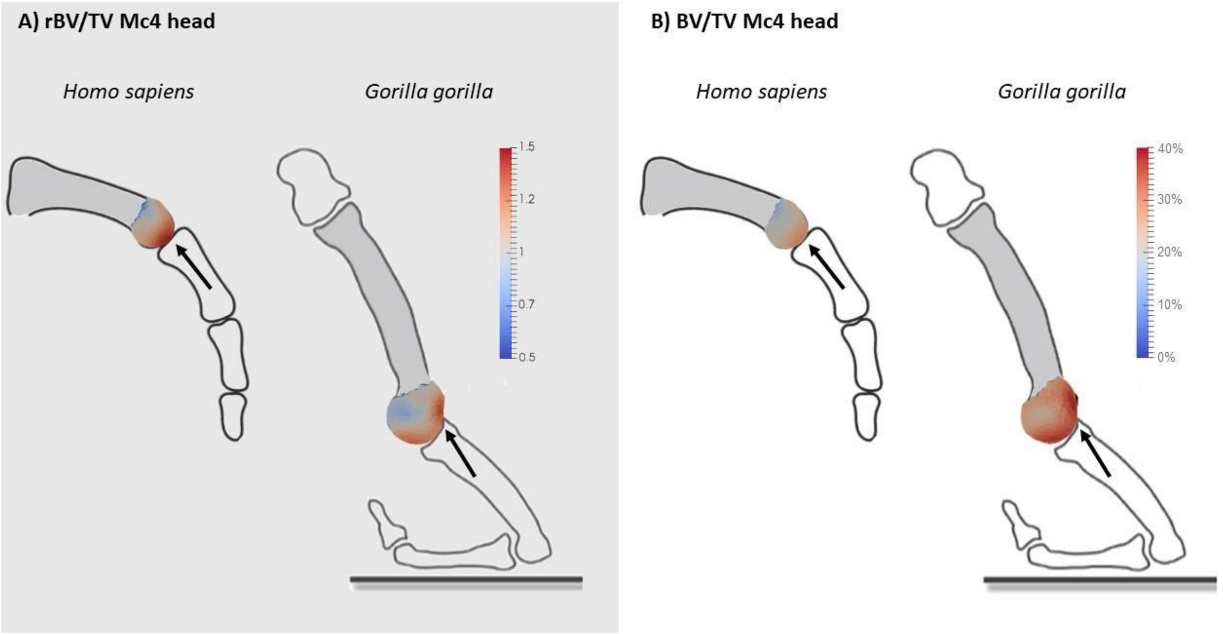

Fig. S17. (A) rBV/TV colormaps (higher values in red, lower in blue) illustrating differences in trabecular distribution in the head of the average fourth metacarpal between humans and gorillas. Gorillas exhibit greater rBV/TV values in the dorsal region of the metacarpal head relative to humans. This pattern is consistent with the distinct habitual joint loading regimes in each species: metacarpophalangeal joints in gorillas are more frequently loaded in hyperextension during knuckle-walking, whereas in humans, they are primarily loaded in flexion, as schematically illustrated in the parasagittal section in ulnar view. (B) Comparison of raw BV/TV values would render this spatial distribution difference less detectable, as gorillas simply have more bone through the entire fourth metacarpal head than humans.

Limitations

This approach nonetheless has some limitations. First, it cannot provide precise joint load directions (orientations) or magnitudes, but only identify regions of relatively higher or lower loading conditions. At this stage, directional measures of trabecular alignment (e.g. using fabric tensors or 3D principal trabecular orientation [PTO], the main direction along which the trabecular struts are preferentially aligned) are not yet implemented in the cHMA workflow. Second, spatial patterns of trabecular bone distribution may result from the complex integration of multiple loading sources, including ground reaction forces, muscle activity, and joint constraints (i.e. shape, ligaments and joint capsule), so that single behaviors cannot always be unequivocally inferred. Third, the method depends heavily on comparisons with extant analogues, which are not ‘frozen ancestors’ but lineages with their own distinct locomotor

repertoires. As a result, unique trabecular bone distribution patterns in fossils that are not represented in the comparative sample cannot yet be fully functionally interpreted. Moreover, *in vivo* experimental validation on mammals is still required to test the sensitivity to detect differences of bone loading histories from trabecular structure. Due to these limitations, **inferences made about joint position must be relatively broad and in this paper we focus on definitions of joint postures that are well-documented in extant African apes and humans, such as hip flexion/extension, knee flexion/extension, and ankle dorsiflexion/plantarflexion. We do not aim to precisely determine load orientation or magnitude (i.e. by reconstructing exact joint angles).**

SI References

1. M. C. H. van der Meulen, G. S. Beaupré, D. R. Carter, Mechanobiologic influences in long bone cross-sectional growth. *Bone* **14**, 635–642 (1993).
2. M. C. H. van der Meulen, M. W. Ashford, B. J. Kiratli, L. K. Bachrach, D. R. Carter, Determinants of femoral geometry and structure during adolescent growth. *J. Orthop. Res.* **14**, 22–29 (1996).
3. C. B. Ruff, A. Walker, E. Trinkaus, Postcranial robusticity in *Homo.* III: Ontogeny. *Am. J. Phys. Anthropol.* **93**, 35–54 (1994).
4. C. B. Ruff, B. Holt, E. Trinkaus, Who's afraid of the big bad Wolff? "Wolff's law" and bone functional adaptation. *Am. J. Phys. Anthropol.* **129**, 484–498 (2006).
5. M. C. H. van der Meulen, D. R. Carter, Developmental mechanics determine long bone allometry. *J. Theor. Biol.* **172**, 323–327 (1995).
6. A. Biewener, N. L. Fazzalari, D. D. Konieczynski, R. V. Baudinette, Adaptive changes in trabecular architecture in relation to functional strain patterns and disuse. *Bone* **19**, 1–8 (1996).
7. R. E. Guldberg, M. Richards, N. J. Caldwell, C. L. Kuelske, S. A. Goldstein, Trabecular bone adaptation to variations in porous-coated implant topology. *J. Biomech.* **30**, 147–153 (1997).
8. A. G. Robling, F. M. Hinant, D. B. Burr, C. H. Turner, Improved bone structure and strength after long-term mechanical loading is greatest if loading is separated into short bouts. *J. Bone Miner. Res.* **17**, 1545–1554 (2002).
9. E. Mittra, C. Rubin, Y.-X. Qin, Interrelationship of trabecular mechanical and microstructural properties in sheep trabecular bone. *J. Biomech.* **38**, 1229–1237 (2005).
10. H. Pontzer *et al.*, Trabecular bone in the bird knee responds with high sensitivity to changes in load orientation. *J. Exp. Biol.* **209**, 57–65 (2006).
11. K. J. Carlson, S. Judex, Increased non-linear locomotion alters diaphyseal bone shape. *J. Exp. Biol.* **210**, 3117–3125 (2007).
12. J. D. Polk, J. Blumenfeld, D. Ahluwalia, Knee posture predicted from subchondral apparent density in the distal femur: An experimental validation. *Anat. Rec.* **291**, 293–302 (2008).
13. M. M. Barak, D. E. Lieberman, J.-J. Hublin, A Wolff in sheep's clothing: Trabecular bone adaptation in response to changes in joint loading orientation. *Bone* **49**, 1141–1151 (2011).
14. K. J. Carlson, D. Marchi, Introduction: Towards refining the concept of mobility, in *Reconstructing Mobility,* K. J. Carlson, D. Marchi, Eds. (Springer US, Boston, MA), pp. 1–11 (2014).
15. P. Christen *et al.*, Bone remodelling in humans is load-driven but not lazy. *Nat. Commun.* **5**, 4855 (2014).
16. M. Cazenave *et al.*, Cortical bone distribution in the femoral neck of *Paranthropus robustus. J. Hum. Evol.* **135**, 102666 (2019).
17. C. J. Dunmore *et al.*, The position of *Australopithecus sediba* within fossil hominin hand use diversity. *Nat. Ecol. Evol.* **4**, 911–918 (2020).
18. E. E. Bird, T. L. Kivell, C. J. Dunmore, M. W. Tocheri, M. M. Skinner, Trabecular bone structure of the proximal capitate in extant hominids and fossil hominins with implications for midcarpal joint loading and the dart-thrower's motion. *Am. J. Biol. Anthropol.* **183**, e24824 (2024).
19. M. Cazenave *et al.*, Inner structural organization of the distal humerus in *Paranthropus* and *Homo. C. R. Palevol* **16**, 521–532 (2017).
20. M. Cazenave *et al.*, Trabecular organization of the proximal femur in *Paranthropus robustus:* Implications for the assessment of its hip joint loading conditions. *J. Hum. Evol.* **153**, 102964 (2021).
21. S. Sukhdeo, J. Parsons, X. M. Niu, T. M. Ryan, Trabecular bone structure in the distal femur of humans, apes, and baboons. *Anat. Rec.* **303**, 129–149 (2020).
22. S. Syeda *et al.*, 12th Annual Meeting of the European Society for the Study of Human Evolution Abstracts, Tübingen, 22–24 September 2022. *PaleoAnthropology* **2022**, 809 (2022).

23. T. L. Kivell, A review of trabecular bone functional adaptation: What have we learned from trabecular analyses in extant hominoids and what can we apply to fossils? *J. Anat.* **228**, 569–594 (2016).
24. M. Cazenave, T. L. Kivell, Challenges and perspectives on functional interpretations of australopith postcrania and the reconstruction of hominin locomotion. *J. Hum. Evol.* **175**, 103304 (2023).
25. M. M. Barak *et al.*, Trabecular evidence for a human-like gait in *Australopithecus africanus. PLoS One* **8**, e77687 (2013).
26. K. J. Carlson, H. Chirchir, B. A. Patel, Subchondral properties of the hominoid distal tibia: An indicator of loading during habitually dorsiflexed ankle postures. *Am. J. Phys. Anthropol.* **159**, 109 (2016).
27. A. Su, *The functional morphology of subchondral and trabecular bone in the hominoid tibiotalar joint* (PhD thesis, 2011).
28. Z. J. Tsegai *et al.*, Trabecular and cortical bone structure of the talus and distal tibia in *Pan* and *Homo. Am. J. Phys. Anthropol.* **163**, 784–805 (2017).
29. M. D. Sockol, D. A. Raichlen, H. Pontzer, Chimpanzee locomotor energetics and the origin of human bipedalism. *Proc. Natl. Acad. Sci. U.S.A.* **104**, 12265–12269 (2007).
30. H. Pontzer, D. A. Raichlen, M. D. Sockol, The metabolic cost of walking in humans, chimpanzees, and early hominins. *J. Hum. Evol.* **56**, 43–54 (2009).
31. T. R. Pickering *et al.*, First articulating os coxae, femur, and tibia of a small, young adult hominin from Member 1 (Hanging Remnant) of the Swartkrans Formation, South Africa. *J. Hum. Evol.* **161**, 104138 (2021).
32. A. Lukova *et al.*, Trabecular architecture of the distal femur in extant hominids. *J. Anat.* **245**, 156–180 (2024).
33. L. Georgiou, T. L. Kivell, D. H. Pahr, M. M. Skinner, Trabecular bone patterning in the hominoid distal femur. *PeerJ* **6**, e5156 (2018).
34. Z. J. Tsegai, M. M. Skinner, D. H. Pahr, J.-J. Hublin, T. L. Kivell, Systemic patterns of trabecular bone across the human and chimpanzee skeleton. *J. Anat.* **232**, 641–656 (2018).
35. R. J. Fajardo, R. Müller, Three-dimensional analysis of nonhuman primate trabecular architecture using micro-computed tomography. *Am. J. Phys. Anthropol.* **115**, 327–336 (2001).
36. L. MacLatchy, R. A. Müller, A comparison of the femoral head and neck trabecular architecture of *Galago* and *Perodicticus* using micro-computed tomography (μCT). *J. Hum. Evol.* **43**, 89–105 (2002).
37. T. M. Ryan, R. A. Ketcham, The three-dimensional structure of trabecular bone in the femoral head of strepsirrhine primates. *J. Hum. Evol.* **43**, 1–26 (2002).
38. T. M. Ryan, R. A. Ketcham, Femoral head trabecular bone structure in two omomyid primates. *J. Hum. Evol.* **43**, 241–263 (2002).
39. T. M. Ryan, G. E. Krovitz, Trabecular bone ontogeny in the human proximal femur. *J. Hum. Evol.* **51**, 591–602 (2006).
40. P. Saparin, H. Scherf, J. Hublin, P. Fratzl, R. Weinkamer, Structural adaptation of trabecular bone revealed by position-resolved analysis of proximal femora of different primates. *Anat. Rec.* **294**, 55–67 (2011).
41. T. M. Ryan, C. N. Shaw, Unique suites of trabecular bone features characterize locomotor behavior in human and non-human anthropoid primates. *PLoS One* **7**, e41037 (2012).
42. T. M. Ryan, C. N. Shaw, Gracility of the modern *Homo sapiens* skeleton is the result of decreased biomechanical loading. *Proc. Natl. Acad. Sci. U.S.A.* **112**, 372–377 (2015).
43. D. A. Raichlen *et al.*, An ontogenetic framework linking locomotion and trabecular bone architecture with applications for reconstructing hominin life history. *J. Hum. Evol.* **81**, 1–12 (2015).
44. T. M. Ryan *et al.*, Human-like hip joint loading in *Australopithecus africanus* and *Paranthropus robustus. J. Hum. Evol.* **121**, 12–24 (2018).

45. L. Georgiou, T. L. Kivell, D. H. Pahr, L. T. Buck, M. M. Skinner, Trabecular architecture of the great ape and human femoral head. *J. Anat.* **234**, 679–693 (2019).
46. L. Georgiou *et al.*, Evidence for habitual climbing in a Pleistocene hominin in South Africa. *Proc. Natl. Acad. Sci. U.S.A.* **117**, 8416–8423 (2020).
47. C. B. Ruff, M. L. Burgess, R. A. Ketcham, J. Kappelman, Limb bone structural proportions and locomotor behavior in A.L. 288-1 ("Lucy"). *PLoS One* **11**, e0166095 (2016).
48. J. C. Ohman, T. J. Krochta, C. O. Lovejoy, R. P. Mensforth, B. Latimer, Cortical bone distribution in the femoral neck of hominoids: Implications for the locomotion of *Australopithecus afarensis. Am. J. Phys. Anthropol.* **104**, 117–131 (1997).
49. K. L. Rafferty, Structural design of the femoral neck in primates. *J. Hum. Evol.* **34**, 361–383 (1998).
50. J. T. Stern, R. L. Susman, Electromyography of the gluteal muscles in *Hylobates, Pongo,* and *Pan:* Implications for the evolution of hominid bipedality. *Am. J. Phys. Anthropol.* **55**, 153–166 (1981).
51. C. B. Ruff, R. Higgins, Femoral neck structure and function in early hominins. *Am. J. Phys. Anthropol.* **150**, 512–525 (2013).
52. M. Kimizuka, H. Kurosawa, T. Fukubayashi, Load-bearing pattern of the ankle joint: Contact area and pressure distribution. *Arch. Orthop. Trauma Surg.* **96**, 45–49 (1980).
53. H. Kura, H. B. Kitaoka, Z.-P. Luo, K.-N. An, Measurement of surface contact area of the ankle joint. *Clin. Biomech.* **13**, 365–370 (1998).
54. S. Millington, M. Grabner, R. Wozelka, S. Hurwitz, J. Crandall, A stereophotographic study of ankle joint contact area. *J. Orthop. Res.* **25**, 1465–1473 (2007).
55. J. M. DeSilva, Functional morphology of the ankle and the likelihood of climbing in early hominins. *Proc. Natl. Acad. Sci. U.S.A.* **106**, 6567–6572 (2009).
56. F. E. Grine, The alpha taxonomy of *Australopithecus* at Sterkfontein: The postcranial evidence. *C. R. Palevol* **18**, 335–352 (2019).
57. F. E. Grine, The alpha taxonomy of *Australopithecus africanus,* in *The Paleobiology of Australopithecus,* K. E. Reed, J. G. Fleagle, R. E. Leakey, Eds. (Springer Netherlands), pp. 73–104 (2013).
58. R. J. Clarke, *Australopithecus* from Sterkfontein Caves, South Africa, in *The Paleobiology of Australopithecus,* K. E. Reed, J. G. Fleagle, R. E. Leakey, Eds. (Springer Netherlands), pp. 105–123 (2013).
59. R. J. Clarke, Early Acheulean with *Homo habilis* at Sterkfontein, in *Hominid Evolution: Past, Present and Future,* E. Delson, Ed. (Alan R. Liss, 1985), pp. 287–298.
60. R. J. Clarke, On some new interpretations of Sterkfontein stratigraphy. *S. Afr. J. Sci.* **90**, 211–214 (1994).
61. K. Kuman, R. J. Clarke, Stratigraphy, artefact industries and hominid associations for Sterkfontein, Member 5. *J. Hum. Evol.* **38**, 827–847 (2000).
62. M. Horn, *Clarifying the stratigraphic boundary between Member 4 and Member 5 of the Sterkfontein Caves, South Africa: A three-dimensional spatial analysis of hominin fossils and stone tools* (MSc thesis, Univ. of the Witwatersrand, 2021).
63. D. J. Stratford, L. Bruxelles, R. J. Clarke, K. Kuman, New stratigraphic interpretations of the fossil and artefact-bearing deposits of the Name Chamber, Sterkfontein. *S. Afr. Archaeol. Bull.* **67**, 159–167 (2012).
64. M. Remis, Effects of body size and social context on the arboreal activities of lowland gorillas in the Central African Republic. *Am. J. Phys. Anthropol.* **97**, 413–433 (1995).
65. D. M. Doran, Ontogeny of locomotion in mountain gorillas and chimpanzees. *J. Hum. Evol.* **32**, 323–344 (1997).
66. R. H. Crompton, W. I. Sellers, S. K. S. Thorpe, Arboreality, terrestriality and bipedalism. *Philos. Trans. R. Soc. Lond. B* **365**, 3301–3314 (2010).

67. A. S. Hammond, In vivo baseline measurements of hip joint range of motion in suspensory and nonsuspensory anthropoids. *Am. J. Phys. Anthropol.* **153**, 417–434 (2014).
68. K. Isler, 3D-kinematics of vertical climbing in hominoids. *Am. J. Phys. Anthropol.* **126**, 66–81 (2005).
69. C. O. Lovejoy, R. S. Meindl, J. C. Ohman, K. G. Heiple, T. D. White, The Maka femur and its bearing on the antiquity of human walking: Applying contemporary concepts of morphogenesis to the human fossil record. *Am. J. Phys. Anthropol.* **119**, 97–133 (2002).
70. C. O. Lovejoy, The natural history of human gait and posture. *Gait Posture* **21**, 113–124 (2005).
71. L. Friedl *et al.*, Femoral neck and shaft structure in *Homo naledi* from the Dinaledi Chamber (Rising Star System, South Africa). *J. Hum. Evol.* **133**, 61–77 (2019).
72. C. O. Lovejoy, K. G. Heiple, Proximal femoral anatomy of Australopithecus. Nature 235, 175–176 (1972).
73. E. H. Harmon, The shape of the early hominin proximal femur. Am. J. Phys. Anthropol. 139, 154–171 (2009).
74. R. L. Susman, D. de Ruiter, C. K. Brain, Recently identified postcranial remains of Paranthropus and Early Homo from Swartkrans Cave, South Africa. J. Hum. Evol. 41, 607–629 (2001).
75. T. R. Pickering, M. Cazenave, R. J. Clarke, A. J. Heile, M. V. Caruana, K. Kuman, D. Stratford, C. K. Brain, J. L. Heaton, First articulating os coxae, femur, and tibia of a small adult Paranthropus robustus from Member 1 (Hanging Remnant) of the Swartkrans Formation, South Africa. J. Hum. Evol. 201, 103647 (2025).
76. J. M. DeSilva, Functional morphology of the ankle and the likelihood of climbing in early hominins. Proc. Natl. Acad. Sci. U.S.A. 106, 6567–6572 (2009).
77. M. Cazenave, J. Braga, A. Oettlé, T. R. Pickering, J. L. Heaton, M. Nakatsukasa, J. F. Thackeray, F. de Beer, J. Hoffman, J. Dumoncel, R. Macchiarelli, Cortical bone distribution in the femoral neck of Paranthropus robustus. J. Hum. Evol. 135, 102666 (2019).
78. C. B. Ruff, R. Higgins, Femoral neck structure and function in early hominins. Am. J. Phys. Anthropol. 150, 512–525 (2013).
79. C. O. Lovejoy, K. G. Heiple, Proximal femoral anatomy of Australopithecus. Nature 235, 175–176 (1972).
80. C. Tardieu, Y. Glard, E. Garron, C. Boulay, J.-L. Jouve, O. Dutour, G. Boetsch, G. Bollini, Relationship between formation of the femoral bicondylar angle and trochlear shape: Independence of diaphyseal and epiphyseal growth. Am. J. Phys. Anthropol. 130, 491–500 (2006).
81. M. A. Frelat, C. N. Shaw, S. Sukhdeo, J.-J. Hublin, S. Benazzi, T. M. Ryan, Evolution of the hominin knee and ankle. J. Hum. Evol. 108, 147–160 (2017).
82. K. D. Hunt, S. E. Dunevant, R. M. Yohler, K. J. Carlson, Femoral bicondylar angles among dry-habitat chimpanzees (Pan troglodytes schweinfurthii) resemble those of humans: Implications for knee function, Australopith sexual dimorphism, and the evolution of bipedalism. J. Anthropol. Res. 77, 303–337 (2021).
83. B. Latimer, J. C. Ohman, C. O. Lovejoy, Talocrural joint in African hominoids: Implications for Australopithecus afarensis. Am. J. Phys. Anthropol. 74, 155–175 (1987).
84. J. M. DeSilva, Z. J. Throckmorton, Lucy's flat feet: The relationship between the ankle and rearfoot arching in early hominins. PLoS One 5, e14432 (2010).
85. B. Zipfel, J. M. DeSilva, R. S. Kidd, K. J. Carlson, S. E. Churchill, L. R. Berger, The foot and ankle of *Australopithecus sediba*. Science 333, 1417–1420 (2011).
86. J. Wolff, *Das Gesetz der Transformation der Knochen (The Law of Bone Remodelling.)* (Springer, Berlin, 1892).
87. S. C. Cowin, *Bone Mechanics Handbook* (CRC Press, 2001).
88. O. M. Pearson, D. E. Lieberman, The aging of Wolff's "law": Ontogeny and responses to mechanical loading in cortical bone. *Am. J. Phys. Anthropol.* **125** (Suppl. 39), 63–99 (2004).

89. J. D. Currey, *Bones: Structure and Mechanics* (Princeton Univ. Press, 2002).
90. M. M. Barak, S. Weiner, R. Shahar, Importance of the integrity of trabecular bone to the relationship between load and deformation of rat femora: An optical metrology study. *J. Mater. Chem.* **18**, 3855–3864 (2008).
91. M. M. Barak, Cortical and trabecular bone modeling and implications for bone functional adaptation in the mammalian tibia. *Bioengineering* **11**, 514 (2024).
92. R. Huiskes, R. Ruimerman, G. H. van Lenthe, J. D. Janssen, Effects of mechanical forces on maintenance and adaptation of form in trabecular bone. *Nature* **405**, 704–706 (2000).
93. C. B. Ruff, B. Holt, E. Trinkaus, Who's afraid of the big bad Wolff? "Wolff's law" and bone functional adaptation. *Am. J. Phys. Anthropol.* **129**, 484–498 (2006).
94. T. Sugiyama, L. B. Meakin, W. J. Browne, G. L. Galea, J. S. Price, L. E. Lanyon, Bones' adaptive response to mechanical loading is essentially linear between the low strains associated with disuse and the high strains associated with the lamellar/woven bone transition. *J. Bone Miner. Res.* **27**, 1784–1793 (2012).
95. R. Oftadeh, M. Perez-Viloria, J. C. Villa-Camacho, A. Vaziri, A. Nazarian, Biomechanics and mechanobiology of trabecular bone: A review. *J. Biomech. Eng.* **137**, 010802 (2015).
96. F. Wang, F. Metzner, G. Osterhoff, L. Zheng, S. Schleifenbaum, The role of bone marrow on the mechanical properties of trabecular bone: A systematic review. *Biomed. Eng. Online* **21**, 80 (2022).
97. M. M. Barak, D. E. Lieberman, J. J. Hublin, A Wolff in sheep's clothing: Trabecular bone adaptation in response to changes in joint loading orientation. *Bone* **49**, 1141–1151 (2011).
98. L. Gabel, A. M. Liphardt, P. A. Hulme, M. Heer, S. R. Zwart, J. D. Sibonga, … S. K. Boyd, Incomplete recovery of bone strength and trabecular microarchitecture at the distal tibia 1 year after return from long duration spaceflight. *Sci. Rep.* **12**, 9446 (2022).
99. C. H. Turner, Three rules for bone adaptation to mechanical stimuli. *Bone* **23**, 399–407 (1998).
100. A. Nazarian, D. von Stechow, D. Zurakowski, R. Müller, B. D. Snyder, Bone volume fraction explains the variation in strength and stiffness of cancellous bone affected by metastatic cancer and osteoporosis. *Calcif. Tissue Int.* **83**, 368–379 (2008). 100
101. H. Pontzer, D. E. Lieberman, E. Momin, M. J. Devlin, J. D. Polk, B. Hallgrimsson, D. M. L. Cooper, Trabecular bone in the bird knee responds with high sensitivity to changes in load orientation. *J. Exp. Biol.* **209**, 57–65 (2006).
102. S. C. Cowin, Wolff's law of trabecular architecture at remodeling equilibrium. (1986).
103. S. A. Goldstein, R. Goulet, D. McCubbrey, Measurement and significance of threedimensional architecture to the mechanical integrity of trabecular bone. *Calcif. Tissue Int.* **53** (Suppl. 1), S127–S133 (1993).
104. A. Odgaard, J. Kabel, B. van Rietbergen, M. Dalstra, R. Huiskes, Fabric and elastic principal directions of cancellous bone are closely related. *J. Biomech.* **30**, 487–495 (1997).
105. M. Stauber, R. Müller, Age-related changes in trabecular bone microstructures: Global and local morphometry. *Osteoporos. Int.* **17**, 616–626 (2006). 105
106. G. Maquer, S. N. Musy, J. Wandel, T. Gross, P. K. Zysset, Bone volume fraction and fabric anisotropy are better determinants of trabecular bone stiffness than other morphological variables. *J. Bone Miner. Res.* **30**, 1000–1008 (2015).
107. F. A. Schulte, D. Ruffoni, F. M. Lambers, D. Christen, D. J. Webster, G. Kuhn, R. Müller, Local mechanical stimuli regulate bone formation and resorption in mice at the tissue level. *PLoS One* **8**, e62172 (2013).
108. K. Manda, S. Xie, R. J. Wallace, F. Levrero-Florencio, P. Pankaj, Linear viscoelasticity– bone volume fraction relationships of bovine trabecular bone. *Biomech. Model. Mechanobiol.* **15**, 1631–1640 (2016).
109. M. J. Jendral, D. R. Korver, J. S. Church, J. J. R. Feddes, Bone mineral density and breaking strength of White Leghorns housed in conventional, modified, and commercially available colony battery cages. *Poult. Sci.* **87**, 828–837 (2008).

110.G. A. Hong, B. W. Tobalske, N. van Staaveren, E. M. Leishman, T. M. Widowski, D. R. Powers, A. Harlander, Hen-durance training—effects of an exercise regimen on laying hen muscle architecture and fracture prevalence. *R. Soc. Open Sci.* **12**, 241191 (2025).
111.S. Lee, Y. A. Shin, J. Cho, D. H. Park, C. Kim, Moderate-intensity exercise preserves bone mineral density and improves femoral trabecular bone microarchitecture in middleaged mice. *J. Bone Metab.* **29**, 103 (2022).
112.Z. Liu, J. Gao, H. Gong, Effects of treadmill with different intensities on bone quality and muscle properties in adult rats. *Biomed. Eng. Online* **18**, 107 (2019).
113.K. Manda, S. Xie, R. J. Wallace, F. Levrero-Florencio, P. Pankaj, Linear viscoelasticity– bone volume fraction relationships of bovine trabecular bone. *Biomech. Model. Mechanobiol.* **15**, 1631–1640 (2016). 113
114.S. M. Uddin, Y. X. Qin, Dynamic acoustic radiation force retains bone structural and mechanical integrity in a functional disuse osteopenia model. *Bone* **75**, 8–17 (2015).
115.J. M. Hughes, E. Gaffney-Stomberg, K. I. Guerriere, K. M. Taylor, K. L. Popp, C. Xu, … M. L. Bouxsein, Changes in tibial bone microarchitecture in female recruits in response to 8 weeks of US Army Basic Combat Training. *Bone* **113**, 9–16 (2018).
116.Z. Ritter, D. Belavy, W. W. Baumann, D. Felsenberg, In vivo bone remodeling rates determination and compressive stiffness variations before, during 60 days bed rest and two years follow up: A micro-FE-analysis from HR-pQCT measurements of the Berlin Bed Rest Study-2. *Acta Astronaut.* **132**, 67–77 (2017).
117.J. P. Saers, Y. Cazorla-Bak, C. N. Shaw, J. T. Stock, T. M. Ryan, Trabecular bone structural variation throughout the human lower limb. *J. Hum. Evol.* **97**, 97–108 (2016).
118.F. A. Schulte, F. M. Lambers, G. Kuhn, R. Müller, In vivo micro-computed tomography allows direct three-dimensional quantification of both bone formation and bone resorption parameters using time-lapsed imaging. *Bone* **48**, 433–442 (2011).
119.T. L. Kivell, A review of trabecular bone functional adaptation: What have we learned from trabecular analyses in extant hominoids and what can we apply to fossils? *J. Anat.* **228**, 569–594 (2016).
120.T. L. Kivell, M. M. Skinner, R. Lazenby, J. J. Hublin, Methodological considerations for analyzing trabecular architecture: An example from the primate hand. *J. Anat.* **218**, 209– 225 (2011). 120
121.Z. J. Tsegai, T. L. Kivell, T. Gross, N. H. Nguyen, D. H. Pahr, J. B. Smaers, M. M. Skinner, Trabecular bone structure correlates with hand posture and use in hominoids. *PLoS One* **8**, e78781 (2013).
122.T. Gross, T. L. Kivell, M. M. Skinner, N. H. Nguyen, D. H. Pahr, A CT-image-based framework for the holistic analysis of cortical and trabecular bone morphology. *Palaeontol. Electron.* (2014).
123.S. Bachmann, C. J. Dunmore, M. M. Skinner, D. H. Pahr, A. Synek, A computational framework for canonical holistic morphometric analysis of trabecular bone. *Sci. Rep.* **12**, 5187 (2022).
124.A; Lukova, C. J. Dunmore, S. Bachmann, A. Synek, D. H. Pahr, T. L. Kivell, M. M. Skinner, Trabecular architecture of the distal femur in extant hominids. *J. Anat.* **245**, 156– 180 (2024).
125.S. Bachmann, D. H. Pahr, A. Synek, Hip joint load prediction using inverse bone remodeling with homogenized FE models: Comparison to micro-FE and influence of material modeling strategy. *Comput. Methods Programs Biomed.* **236**, 107549 (2023). 125
126.G. Bergmann, A. Bender, J. Dymke, G. Duda, P. Damm, Standardized loads acting in hip implants. PLoS ONE 11, e0155612 (2016).
127.C. J. Dunmore, S. Bachmann, A. Synek, D. H. Pahr, M. M. Skinner, T. L. Kivell, The deep trabecular structure of first metacarpals in extant hominids. *Am. J. Biol. Anthropol.* **183**, e24695 (2024).

128.B. J. Addison, D. E. Lieberman, Assessing patterns of variation in BV/TV in the calcaneus and C2 vertebra of *Gorilla gorilla*, *Pan troglodytes*, and populations of *Homo sapiens* from the Pleistocene and Holocene that differ in physical activity levels. *Am. J. Phys. Anthropol.* **173**, 337–349 (2020).
129.L. Georgiou *et al*., Evidence for habitual climbing in a Pleistocene hominin in South Africa. *Proc. Natl. Acad. Sci. U.S.A.* **117**, 8416–8423 (2020).
130.M. Cazenave, A. Oettlé, T. R. Pickering, J. L. Heaton, M. Nakatsukasa, J. F. Thackeray, R. Macchiarelli, Trabecular organization of the proximal femur in *Paranthropus robustus*: Implications for the assessment of its hip joint loading conditions. *J. Hum. Evol.* **153**, 102964 (2021). 130
131.H. Chirchir, T. L. Kivell, C. B. Ruff, J.-J. Hublin, K. J. Carlson, B. Zipfel, B. G. Richmond, Recent origin of low trabecular bone density in modern humans. *Proc. Natl. Acad. Sci. U.S.A.* **112**, 366–371 (2015).
132.T. M. Ryan, C. N. Shaw, Gracility of the modern *Homo sapiens* skeleton is the result of decreased biomechanical loading. *Proc. Natl. Acad. Sci. U.S.A.* **112**, 372–377 (2015).
133.D. E. Lieberman, How and why humans grow thin skulls: Experimental evidence for systemic cortical robusticity. *Am. J. Phys. Anthropol.* **101**, 217–236 (1996). 133
134.S. Sukhdeo, J. Parsons, X. M. Niu, T. M. Ryan, Trabecular bone structure in the distal femur of humans, apes, and baboons. Anat. Rec. 303, 129–149 (2020).
135.A. D. Sylvester, C. E. Terhune, Trabecular mapping: Leveraging geometric morphometrics for analyses of trabecular structure. Am. J. Phys. Anthropol. 163, 553–569 (2017).
136.L. J. DeMars, N. B. Stephens, J. P. Saers, A. Gordon, J. T. Stock, T. M. Ryan, Using point clouds to investigate the relationship between trabecular bone phenotype and behavior: An example utilizing the human calcaneus. Am. J. Hum. Biol. 33, e23468 (2021).
137.C. M. Harper, B. A. Patel, Trabecular bone variation in the gorilla calcaneus. Am. J. Biol. Anthropol. 184, e24939 (2024).
138.M. C. H. van der Meulen, G. S. Beaupré, D. R. Carter, Mechanobiologic influences in long bone cross-sectional growth. Bone 14, 635–642 (1993).
